# Discordance in STING-Induced Activation and Cell Death Between Mouse and Human Dendritic Cell Populations

**DOI:** 10.3389/fimmu.2022.794776

**Published:** 2022-02-25

**Authors:** Ee Shan Pang, Ghazal Daraj, Katherine R. Balka, Dominic De Nardo, Christophe Macri, Hubertus Hochrein, Kelly-Anne Masterman, Peck S. Tan, Angus Shoppee, Zoe Magill, Nazneen Jahan, Mariam Bafit, Yifan Zhan, Benjamin T. Kile, Kate E. Lawlor, Kristen J. Radford, Mark D. Wright, Meredith O’Keeffe

**Affiliations:** ^1^ Department of Biochemistry and Molecular Biology, Biomedicine Discovery Institute, Monash University, Clayton, VIC, Australia; ^2^ Translational Research Institute, Mater Research-University of Queensland, Woolloongabba, QLD, Australia; ^3^ Department of Anatomy and Developmental Biology, Biomedicine Discovery Institute, Monash University, Clayton, VIC, Australia; ^4^ Department of Research, Bavarian Nordic GmbH, Martinsried, Germany; ^5^ Immunology Division, The Walter and Eliza Hall Institute of Medical Research, Parkville, VIC, Australia; ^6^ Department of Medical Biology, University of Melbourne, Parkville, VIC, Australia; ^7^ Faculty of Health and Medical Sciences, The University of Adelaide, Adelaide, SA, Australia; ^8^ Centre for Innate Immunity and Infectious Diseases, Hudson Institute of Medical Research, Clayton, VIC, Australia; ^9^ Department of Molecular and Translational Science, Monash University, Clayton, VIC, Australia; ^10^ Department of Immunology and Pathology, Monash University, Melbourne, VIC, Australia

**Keywords:** STING activation, dendritic cell (DC), interferon-lambda, human dendritic cells, type III interferons, cell death, plasmacytoid dendritic cells (pDCs)

## Abstract

Stimulator of Interferon Genes (STING) is a cytosolic sensor of cyclic dinucleotides (CDNs). The activation of dendritic cells (DC) *via* the STING pathway, and their subsequent production of type I interferon (IFN) is considered central to eradicating tumours in mouse models. However, this contribution of STING in preclinical murine studies has not translated into positive outcomes of STING agonists in phase I & II clinical trials. We therefore questioned whether a difference in human DC responses could be critical to the lack of STING agonist efficacy in human settings. This study sought to directly compare mouse and human plasmacytoid DCs and conventional DC subset responses upon STING activation. We found all mouse and human DC subsets were potently activated by STING stimulation. As expected, Type I IFNs were produced by both mouse and human plasmacytoid DCs. However, mouse and human plasmacytoid and conventional DCs all produced type III IFNs (i.e., IFN-λs) in response to STING activation. Of particular interest, all human DCs produced large amounts of IFN-λ1, not expressed in the mouse genome. Furthermore, we also found differential cell death responses upon STING activation, observing rapid ablation of mouse, but not human, plasmacytoid DCs. STING-induced cell death in murine plasmacytoid DCs occurred in a cell-intrinsic manner and involved intrinsic apoptosis. These data highlight discordance between STING IFN and cell death responses in mouse and human DCs and caution against extrapolating STING-mediated events in mouse models to equivalent human outcomes.

## Introduction

Stimulator of IFN genes (STING, also known as TMEM173, MITA, MPYS or ERIS) is a pattern recognition receptor (PRR) that recognises cytosolic DNA in the form of cyclic dinucleotides (CDNs), such as the bacterial product cyclic-guanosine monophosphate-adenosine monophosphate (3’3’ cGAMP) (1–4). In addition to bacterial components, other forms of DNA from viruses, or the host cell, that find their way into the cytosol are recognised by an enzyme c-GMP-AMP (cGAMP) synthase (cGAS). Upon cytosolic DNA binding, cGAS converts ATP and GTP into the metazoan-specific CDN 2’3’-cGAMP for STING recognition and activation (4–6). STING is a transmembrane protein that exists as dimers anchored within the endoplasmic reticulum membrane and forms a V-shaped pocket that enables cytosolic CDN binding. Ligand binding results in significant conformational changes in the C-terminal domain of STING, mediating its transport to Golgi compartments. At the Golgi, STING recruits TANK-binding kinase 1 (TBK1), which facilitates IRF3 phosphorylation, nuclear translocation and the strong induction of transcription of type I IFNs (e.g. IFN-β) (7–12). STING also triggers a robust pro-inflammatory cytokine response [e.g. tumour necrosis factor (TNF)] by activating Nuclear Factor-kappa B (NF-κB) and this part of the pathway can be mediated independent of TBK1 *via* a closely related homologue protein, IKKϵ (13).

STING activation, and associated type I IFN responses, are required for optimal immune responses to infectious pathogens and DNA-based vaccines (14), as well as for anti-tumour responses (15), including after DNA damage induced by radiation and chemotherapies (16–18). Thus, therapeutics directly targeting STING represent an important avenue to explore. Indeed, in mouse, the use of STING agonists has emerged as a powerful tool to eliminate tumours, both when used alone or as an adjunct to enhance checkpoint immunotherapies (15, 19–21). However, human clinical trials to date have failed to recapitulate the promising pre-clinical responses observed in animal models (22, 23). This lack of efficacy has been somewhat surprising as previous studies have suggested that STING-induced signaling pathways that lead to type I IFN and pro-inflammatory cytokine production are intact in humans and mice (13).

Dendritic cells (DCs) are important players in innate immune responses and their activation is required for the induction of specific immunity. It has been proposed that a DC type I IFN (IFN-α, β) response is essential for STING-mediated immune responses (14, 24). The current dogma garnered from mouse tumour models proposes that DC ingestion of tumour DNA, or use of STING agonist therapy, leads to STING-dependent IFN-β production that enhances conventional (c) DC function to boost subsequent anti-tumour immune responses (15, 24). However, any further detailed molecular studies focused on STING activation in cDCs have largely focused on monocyte-derived DCs generated *in vitro* with GM-CSF (9). A direct comparison of human and mouse DC responses to STING activation is therefore of critical importance, but to date has not been carried out.

DCs are categorised into different subsets with specialised functions and are conserved across species. Plasmacytoid DCs (pDCs), the original natural interferon producing cells (NIPC), are major type I IFN producers. The cDC1 subset excels at the presentation of exogenous antigen on MHCI (cross-presentation), particularly when associated as particulate or cell-associated antigen, and cDC1 are excellent at inducing and activating cytotoxic T cells (CTL). cDC2s are superior at enhancing CD4^+^ T cell activation through MHCII presentation and are important for inducing T helper 2 (Th2) and Th17 cellular responses [reviewed in (25)]. We have previously shown that pDCs and cDC1s also have the specialized capacity to produce high levels of Type III IFNs (or IFN-λ) in response to TLR7/9 and TLR3 ligands, respectively, and to multiple viruses (26). Others have also recently shown that human pDCs produce IFN-λ in response to STING agonists, similar to TLR7 or 9 activation (27).

The IFN-λ family of genes differs between human and mouse in that the mouse genome encodes only 2 highly homologous genes, IFN-λ2 and IFN-λ3, whilst the human genome universally encodes 3 genes, IFN-λ1, -λ2 and -λ3. Moreover, polymorphisms in the IFN-λ gene locus that are more prevalent in people of African descent (28, 29) can lead to expression of a fourth IFN-λ gene, IFN-λ4, which exhibits only approximately 40% identity to the other IFN-λ genes but still signals through the IFN-λ Receptor (R) (30). IFN-λ proteins bind to the IFN-λR composed of the unique IFNLR1 (IL-28RA) and the IL-10Rβ. The IFN-λR, although distinct from the type I IFNR, similarly employs JAK-STAT signaling leading to the transcription of hundreds of interferon stimulated genes (ISGs) and antiviral activity (31). However, IFN-λR expression is mainly confined to epithelial cells and select subsets of hematopoietic cells, including DC, with much work still to be done to refine expression during health and disease settings (32). Overall, IFN-λ seems to have evolved to protect mucosal and epithelial barriers. Whilst IFN-λ1 and -λ2 induce upregulation of similar sets of ISGs, there is some evidence that IFN-λ1 lacks gene repressor function compared to IFN-λ2 (33). Whether unique function of IFN-λ1 in specific epithelial and hematopoietic cells generates differences in overall IFN-λ signaling outcomes in mouse versus humans is not yet known. In addition, expression of IFN-λ4, together with other polymorphisms within the IFN-λ locus, is strongly linked to poor clearance of Hepatitis C Virus (34). Polymorphisms within the IFN-λ gene locus have also recently been linked with the incidence and/or mortality of other diseases including liver inflammation, fibrosis and non-alcoholic fatty liver disease and certain cancers (28, 29, 35). Intriguingly, the mechanisms behind how these polymorphisms affect viral infection, inflammatory disease and cancer, as well as the involvement of high IFN-λ producing cells such as DCs in these contexts, are as yet unclear.

Here we present the first detailed comparison of STING activation in freshly isolated *ex vivo* DCs from mice and humans, and reveal divergent molecular and cellular responses. STING ligation by cyclic dinucleotides (CDNs) markedly upregulated cell surface expression markers of DC activation and maturation to levels at least as prominent as TLR activation. Moreover, STING activation elicited equivalent cell death in human blood and mouse cDC2. In contrast, while mouse pDC were rapidly ablated, human pDCs were refractory to STING-induced killing. Importantly, in response to STING stimulation, differential IFN production was observed by DC subsets that is shared across the species. pDC of both species produced IFN-α, whilst all DC subsets produce IFN-2 and -3 proteins upon STING activation. IFN-λ1 was the most highly expressed IFN from all human DC subsets. This work places IFN-λ in the spotlight as a potential major player in DC-mediated immune responses downstream of STING activation.

## Results

### DC Subsets Reveal Differential Signaling Upon STING Activation but IFN-λ Is Produced From All Mouse DC Subsets

**Figure 1:**
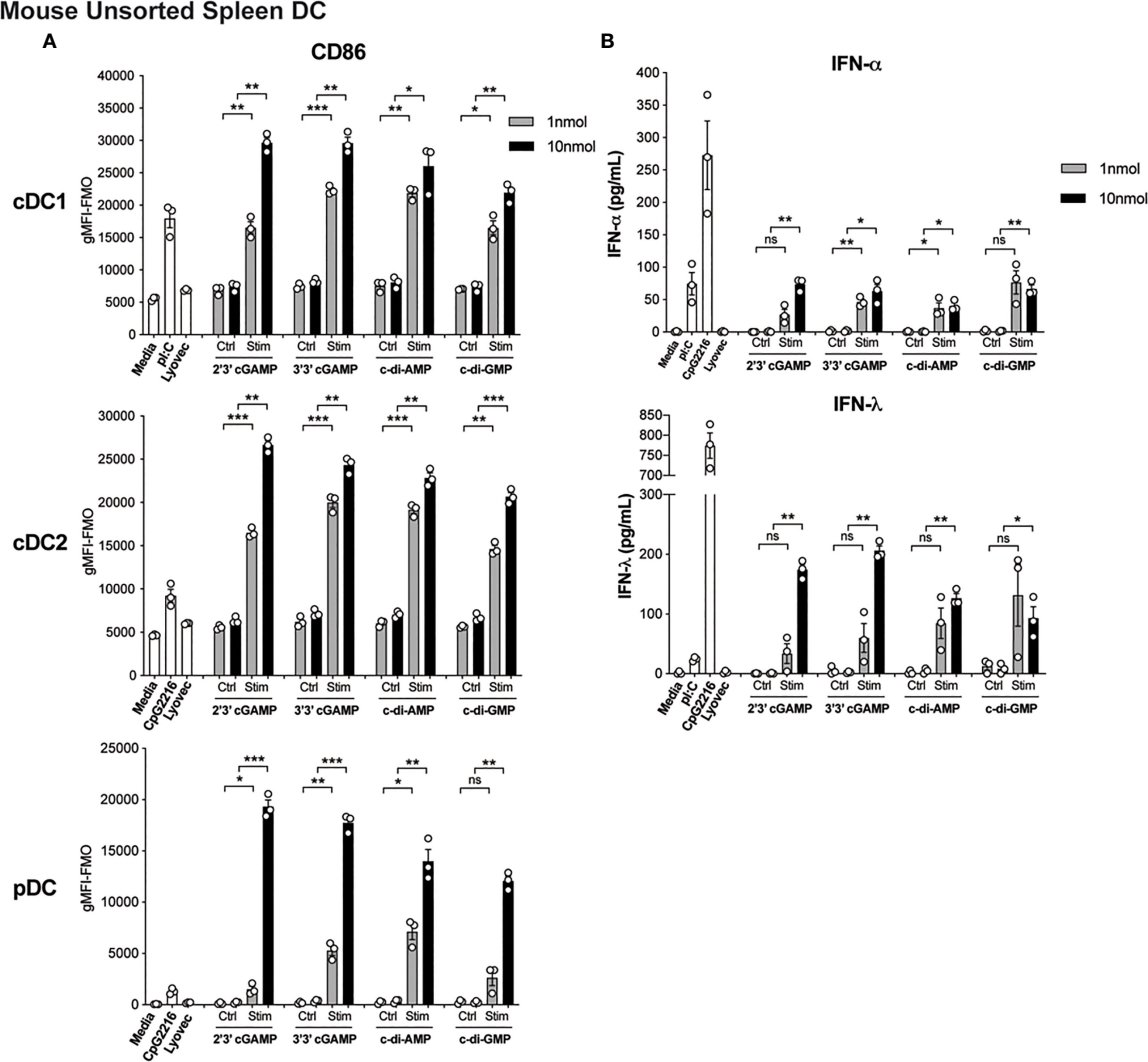
Triggering STING with CDNs induces potent activation and IFN production in mouse DCs. Bulk splenic mouse DCs were stimulated with 1 or 10 nmol 2’3’ cGAMP, 3’3’ cGAMP, c-di-AMP or c-di-GMP complexed with lyovec, their respective linearized control ligands (Ctrl) complexed with lyovec, lyovec alone, 0.5 μM CpG2216 or 100 μg/mL pI:C for 18 h. **(A)** CD86 expression on DC subsets was determined using flow cytometry. Bar graphs represent the mean difference between geometric mean fluorescence intensities (gMFI) of stained samples and fluorescence minus one (FMO) controls ± SEM from 3 biological replicates (pool of 2 mice per replicate). **(B)** IFN production in cell culture supernatants were analysed by ELISA. Bar graphs represent mean ± SEM from 3 biological replicates (pool of 2 mice per replicate). Statistical analyses were performed using two-tailed Paired Student’s *t* test where *P < 0.05, **P < 0.01, ***P < 0.001 and ns, not significant.

The activation of STING in cDCs is reported to lead to type I IFN production (14), enabling enhanced T cell stimulation and therefore is likely to be essential for co-ordinating downstream T cell-mediated immune responses. However, the direct effects of STING activation on mouse and human DC function is less clear but remains of immense interest to understand the potential adjuvant properties of STING ligands. We therefore firstly tested the ability of an array of cyclic dinucleotide (CDN) STING ligands, c[G(3’,5’)pA(3’,5’)p] (3’3’ cGAMP), 2’3’ cGAMP, c di-AMP, c di-GMP, versus linearised controls, to activate and induce type I and III IFN production in mouse splenic DC cultures. All STING ligands led to upregulation of CD86 (**Figure 1A**) and MHCII (**Supplementary Figure 1A** and **Supplementary Figure 2**) on pDC and cDC subsets. In fact, cGAMP (10 nmol) induced CD86 expression on DC, most notably on pDC, that far surpassed the level seen in response to TLR activation (**Figure 1A**). Analyses of IFNs in these cultures also revealed that upon activation, STING induced appreciable levels of IFN-α and IFN-λ (**Figure 1B**), albeit at lower levels than the TLR9 ligand, CpG-A (CpG 2216). Importantly, IFN production was abrogated in DC lacking STING (**Supplementary Figure 1B**), confirming the responses were indeed STING-dependent. However, whether cell activation and IFN production was intrinsic to each of the DC subsets examined, or a bystander event upon selective DC activation, remained unclear.

**Figure 2:**
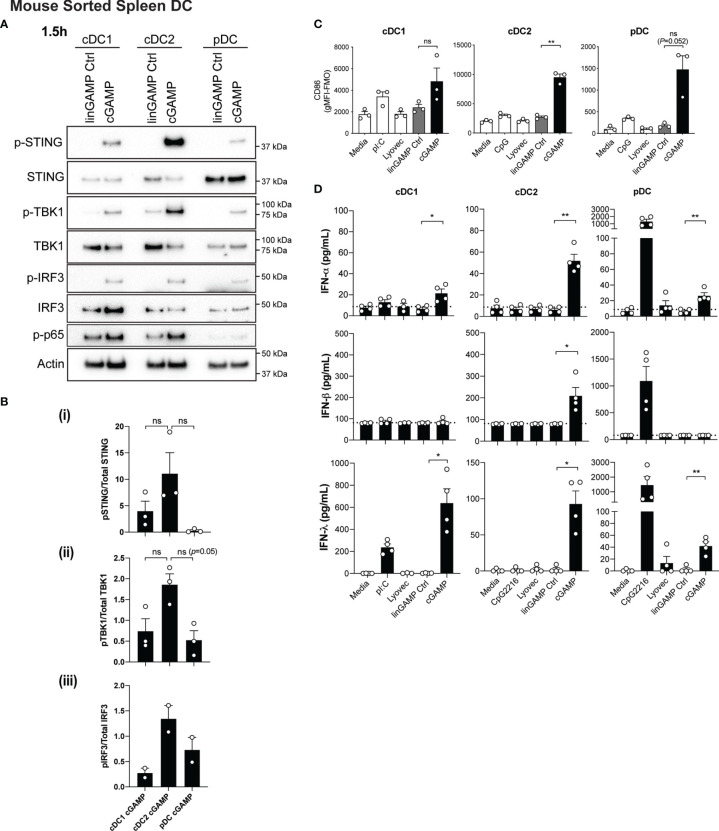
Type I and III IFNs are differentially produced by DC subsets after cGAMP stimulation. **(A, B)** Sorted splenic mouse cDC1, cDC2 and pDCs (see **Supplementary Figure 4A** for sorting strategy) from a pool of 11-12 mice were stimulated with 10 nmol 3’3’ cGAMP or its linearized control ligand (linGAMP Ctrl) complexed with lyovec for 1.5 hrs. **(A)** Cells were then lysed and blotted using the indicated antibodies. Data shown represents 1 of 2-3 independent experiments. **(B)** Densitometric analysis of (i) phosphorylated STING relative to total STING (ii) phosphorylated TBK1 relative to total TBK1 and (iii) phosphorylated IRF3 relative to total IRF3 immunoblots from 2-3 independent experiments are shown. Bar graphs show mean ± SEM. **(C)** Sorted splenic mouse cDC1, cDC2 and pDCs from a pool of 15-17 mice were stimulated with 10 nmol 3’3’ cGAMP or its linearized control ligand (linGAMP Ctrl) complexed with lyovec, lyovec alone, 0.5 μM CpG2216 or 100 μg/mL pI:C for 18 h. CD86 expression on DC subsets was determined using flow cytometry. Bar graphs represent the mean difference between geometric mean fluorescence intensities (gMFI) of stained samples and fluorescence minus one (FMO) controls ± SEM from 3 independent experiments. **(D)** Sorted splenic mouse cDC1, cDC2 and pDCs from a pool of 15-17 mice were stimulated with 10 nmol 3’3’ cGAMP or its linearized control ligand (linGAMP Ctrl) complexed with lyovec, lyovec alone, 0.5 μM CpG2216 or 100 μg/mL pI:C in the presence of 0.2 ng/mL GM-CSF for 18 hrs. IFN production in cell culture supernatants was analysed by ELISA (IFN-α and IFN-λ) or flow cytometric bead assay (IFN-β). Bar graphs represent mean ± SEM from 4 independent experiments. Statistical analyses were performed using two-tailed Paired Student’s *t* test where *P < 0.05, **P < 0.01 and ns, not significant.

Conventional DC1 (cDC1) activation has been observed *in vivo* in response to STING-activating adjuvants (36). Moreover, activation of cDC1 through STING is purported to be critical in inducing CTL responses upon immunotherapy administration (reviewed in (37)). However, in these scenarios, whether cDC1 *directly* respond to STING ligands has not been examined in detail. We therefore firstly examined levels of STING in DC subsets and found that STING (encoded by the gene *Tmem173*) is differentially expressed amongst DC subsets with expression in mouse pDC >cDC2 >cDC1 (**Supplementary Figure 3A**). Next, to determine the response of individual DC subsets to STING ligands, we sorted freshly isolated mouse splenic DCs into cDC1 (CD11c^hi^CD317^lo^CD8^+^CD11b^-^), cDC2 (CD11c^hi^CD317^lo^CD8^-^CD11b^+^) and pDC (CD11c^int^CD317^hi^CD11b^-^) subsets (**Supplementary Figure 4A**), and stimulated them with 3’3’-cGAMP for 1.5 hours. Consistent with our gene expression data (**Supplementary Figure 3A**), Western blotting confirmed differential protein levels of total STING between DC subsets. These analyses further indicated that the cDC1 and cDC2 populations showed similar signaling events within the STING signaling pathway, with activation of NF-κB-p65, TBK1, IRF3 and STING (**Figures 2A, B**). This was commensurate with signaling events shown for other cell types (13); with the exception that we were unable to detect IKKϵ protein in any DC subset (not shown). Although not reaching significance, there was a trend for cDC2 to express the highest relative level of phospho (P)-STING, and the phosphorylated form of the STING-activating kinase, TBK1 (**Figures 2A, B**). Intriguingly, despite pDC exhibiting the highest levels of total STING protein they demonstrated modest STING phosphorylation and IRF3 activation, and showed little to no activation of NF-κB p65 (**Figures 2A, B**). These data strongly suggest differential regulation of the STING signaling pathways between cDCs and pDCs.

In view of the fact that all DC subsets were STING competent, we next examined the expression of co-stimulatory markers CD86 and CD80 in cultured DC subsets by FACS (**Figure 2C** and **Supplementary Figure 5**). It is worth noting that we and others routinely observe that isolated cDCs upregulate these activation markers *in vitro* in media alone (38) and that further increases are observed in cDC1 and cDC2 upon TLR3 (poly I:C) or TLR9 (CpG) activation, respectively (**Figure 2C**). Remarkably, we observed that 3’3’-cGAMP stimulation potently induced significant upregulation of CD86 (**Figure 2C**) and CD80 expression in cDC2 (**Supplementary Figure 5**), commensurate with increased STING signaling responses (**Figure 2A**). Compared to the negative controls, this upregulation was notably higher than the CD86 and CD80 expression elicited by CpG stimulation (**Figure 2C** and **Supplementary Figure 5**). Likewise, pDCs, which normally exhibit modest upregulation of CD86 and CD80 on their surface after CpG-induced activation, also expressed heightened expression of these markers after cGAMP stimulation (**Figure 2C** and **Supplementary Figure 5**). This is perhaps surprising given the relatively poor STING phosphorylation observed in pDCs upon stimulation (**Figure 2A**). Commensurate with the low levels of STING we observed in cDC1, cGAMP only slightly enhanced CD86 (**Figure 2C**) and CD80 (**Supplementary Figure 5**) levels, which were more comparable to that induced by poly I:C (**Figure 2C** and **Supplementary Figure 5**).

We have previously documented that cDC1 and pDCs produce not only large quantities of type I IFNs but also IFN-λ in response to poly I:C and CpG stimulation, respectively (26). Consequently, we tested supernatants from sorted mouse DC subsets treated with 3’3’-cGAMP for production of IFN-α and -β, as well as IFN-λ, by ELISA and bead array. In order to support the viability of sorted cDC after sorting and maximise IFN-λ production (26), we included a low concentration of GM-CSF (0.2 ng/ml) in the stimulated cultures. Interestingly, the levels of STING activation in each of the DC subsets was not directly related to levels of IFN production (**Figures 2A, D**). Consistent with their specialised IFN-I producing function, pDCs produced low levels of IFN-α in response to cGAMP stimulation (**Figure 2D**). However, cDC2 and cDC1 also produced low levels of IFN-α that was only detectable in the presence of GM-CSF (**Figure 2D** and **Supplementary Figure 6**). The major producers of IFN-β were the cDC2 subset, while IFN-λ2/3 was produced by *all* DC subsets in response to 3’3’ cGAMP–mediated STING activation (**Figure 2D**), with cDC1 producing the most, most prominently when supplemented with GM-CSF (**Figure 2D** and **Supplementary Figure 6**). Overall these data reveal that while all DC subsets activate STING upon CDN treatment they differ in their downstream signaling and IFN responses.

### STING-Dependent Activation Occurs Co-Incident With Potent Killing of Mouse pDCs

**Figure 3:**
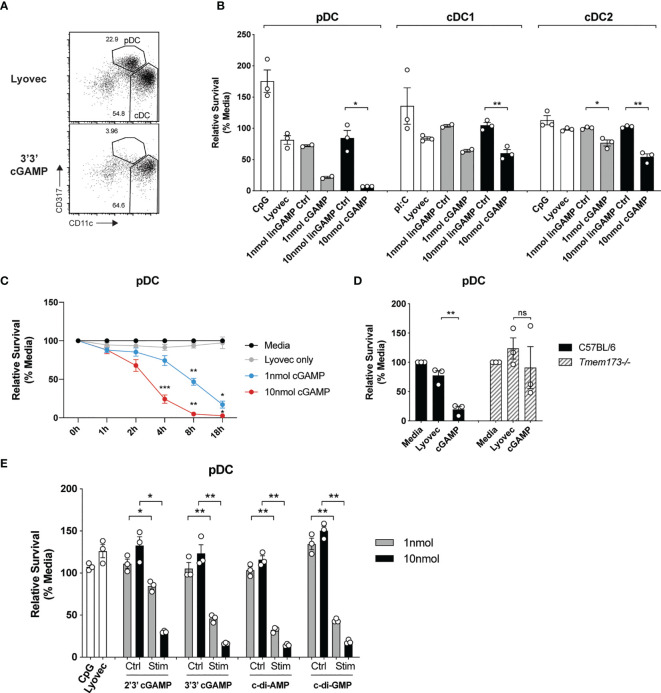
cGAMP stimulation induces rapid, potent killing of mouse pDCs. **(A)** FACS plots showing bulk mouse splenic pDCs (CD11c^int^CD317^hi^) and cDCs (CD11c^hi^CD317^lo^) stimulated for 18 h with 10 nmol 3’3’ cGAMP complexed with lyovec, or lyovec alone. **(B)** Sorted mouse splenic pDCs and cDC subsets from a pool of 15-17 mice were stimulated for 18 h with 1 or 10 nmol 3’3’ cGAMP, its linearized control ligand (linGAMP Ctrl) complexed with lyovec, lyovec alone, 0.5 μM CpG2216 or 100 μg/mL pI:C. Bar graphs show the mean relative survival (compared to media alone) ± SEM compiled from 2-3 independent experiments. **(C)** Bulk splenic DCs from a pool of 5-6 mice per replicate were stimulated with 1 or 10 nmol 3’3’ cGAMP complexed with lyovec or lyovec alone for the indicated time points. Line graph depicts the mean relative survival (compared to media alone) ± SEM combined from 3 independent experiments. **(D)** Bulk splenic DCs from C57BL/6 or *Tmem173^-/-^* mice were stimulated with 10 nmol 3’3’ cGAMP complexed with lyovec or lyovec alone for 18 h. Bar graphs show their mean pDC relative survival ± SEM from 3 individual mice per genotype. **(E)** Bulk splenic DCs were stimulated with 1 or 10nmol 2’3’ cGAMP, 3’3’ cGAMP, c-di-AMP or c-di-GMP complexed with lyovec, their respective linearized control ligands (Ctrl) complexed with lyovec, lyovec alone or 0.5 μM CpG2216 for 18 h. Bar graphs represent the mean relative survival of pDCs ± SEM from 3 biological replicates (pool of 2 mice per replicate). Statistical analyses were performed using **(B, D, E)** two-tailed Paired Student’s *t* test or **(C)** two-way ANOVA using Tukey’s test to correct for multiple comparisons where *P < 0.05, **P < 0.01, ***P < 0.001 and ns, not significant.

It has recently been recognized that STING signaling can trigger multiple forms of cell death, including apoptosis, pyroptosis and necroptosis (39), but the effects on DCs are unknown. Given the potential importance of antigen presentation and IFN responses induced in individual DC subsets after STING activation, we set out to determine whether STING signaling induced death of DC subsets. Analyses of the total spleen mouse DCs after activation with CDNs revealed that pDC numbers were obliterated compared to stimulation with a control linearized dinucleotide ligand (**Figure 3A**). The analyses of sorted spleen DCs revealed this cGAMP-induced death was intrinsic to the pDC and not due to feedback from the other DCs in the total DC cultures (**Figure 3B**). This was in stark contrast to TLR9 activation with CpG, which enhanced survival of pDCs compared to media alone (**Figure 3B**). In addition to the depletion of pDCs, we further observed a significant reduction in sorted cDC after stimulation with cGAMP, although to a lesser extent (**Figure 3B**) and cDC1 and cDC2 population ratios remained similar in the unsorted DC (**Supplementary Figure 7**). Remarkably, pDC cell death was rapid (significant from 4 hours onwards, **Figure 3C**) and stimulation with a 10-fold lower concentration of cGAMP (1nmol) still induced killing of pDCs, albeit reduced (**Figure 3C**). These results suggest murine pDCs are more sensitive to cell death upon STING activation than both cDC1 and cDC2s.

To determine the specificity of STING in inducing rapid pDC death we assessed pDCs viability upon the genetic deletion of STING. STING deficiency (*Tmem173^-/-^*) completely protected pDCs from cell death following 3’3’ cGAMP stimulation (**Figure 3D**) thereby confirming this is a STING-dependent event. Given that different CDNs bind to STING with different affinities and could potentially affect STING activation (40), we next determined whether other CDNs were able to similarly induce DC death in mixed cultures of spleen DC. Significant pDC death occurred after stimulation with all four types of CDNs tested, although bacterial CDNs (3’3’ cGAMP, c-di-AMP, c-di-GMP) were more potent at inducing cell death when compared to mammalian 2’3’ cGAMP at a lower (1 nmol) dose (**Figure 3E**). For cDCs, the cDC2 subset exhibited increased death to all CDNs over the controls (**Supplementary Figure 8**), however, a drop in the cDC1 population numbers was only observed after c-di-AMP stimulation (**Supplementary Figure 8**). Overall, cell death in these mixed cultures (**Supplementary Figure 8**) was not as dramatic as seen in the sorted populations (**Figure 3B**). We did not further investigate the reason for this but propose that a combination of cell:cell crosstalk and lack of stress induced by cell sorting slightly enhanced the survival of the unsorted populations.

### Soluble Mediators Induced by STING Activation Are Not Responsible for Mouse pDC Death

**Figure 4:**
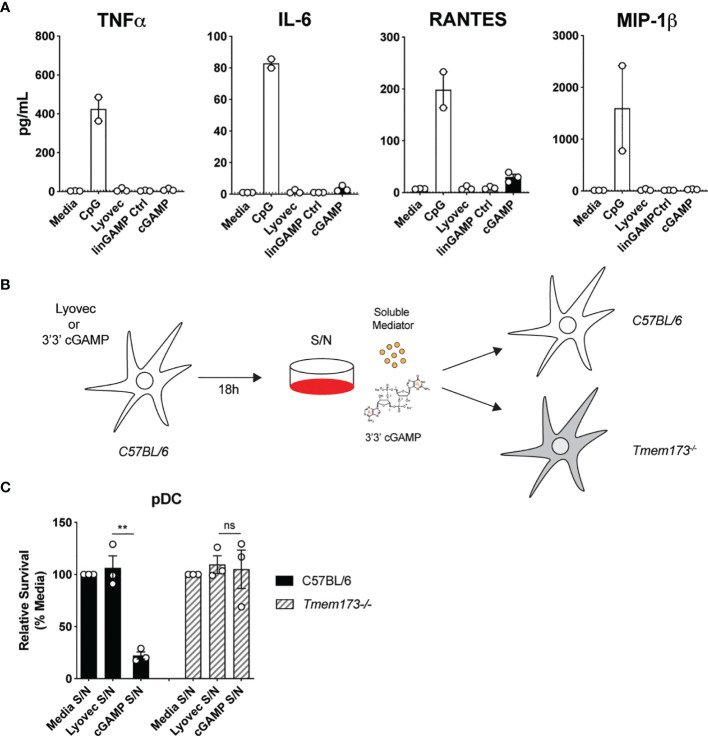
Soluble mediators induced by STING activation are not responsible for pDC death. **(A)** Sorted splenic pDC from a pool of 15-17 mice were stimulated for 18 hrs with 10 nmol 3’3’ cGAMP or its linearized control ligand (linGAMP Ctrl) complexed with lyovec, lyovec alone or 0.5 μM CpG1668. Cytokine and chemokine production in supernatants were measured by flow cytometric bead assay. Bar graphs depict data from 2-3 independent experiments showing mean ± SEM. **(B, C)** Supernatants harvested 18 h after bulk C57BL/6 DCs were stimulated with 10 nmol 3’3’ cGAMP complexed with lyovec, lyovec alone or media were diluted 1/5 in media and were used to stimulate freshly isolated DCs from C57BL/6 or *Tmem173^-/-^* mice for 18 h. **(B)** Schematic diagram showing experimental design. **(C)** Bar graphs show mean relative survival (compared to media supernatant alone) ± SEM from 3 individual mice per genotype. Statistical analysis was performed using two-tailed Paired Student’s *t* test where **P < 0.01 and ns, not significant.

To clarify the mode of pDC death after STING activation, we first investigated if pDCs were dying *via* a direct, intrinsic mechanism following STING activation, or if they were producing a soluble factor that could feedback on cells and induce cell death extrinsically. Some cytokines, such as the key pro-inflammatory cytokine TNF, can bind to receptors that have death domains and trigger apoptosis (41). Therefore, we examined the cytokine and chemokine profile in the supernatants of mouse pDCs stimulated with cGAMP. Although pDCs produced large quantities of TNF, IL-6, RANTES and MIP-1β in response to CpG stimulation, they produced much lower amounts of these cytokines and chemokines after cGAMP stimulation (**Figure 4A**). Others have shown that IL-10, previously implicated in human pDC death (42), can also be produced in response to STING activation (43). We did not detect any IL-10 in the supernatants of pDCs stimulated with cGAMP (data not shown). However, other types of soluble mediators not tested here, including hormones, could be present in these supernatants and induce pDC death after STING activation (44).

To formally address if pDC death could be induced by a soluble factor produced after STING activation, we stimulated splenic DCs from wild-type (WT) C57BL/6 mice with 3’3’ cGAMP/lyovec complexes then harvested the culture supernatants after 18 hours to isolate potential soluble death-inducing factors. Freshly isolated DCs from WT and *Tmem173^–/–^* mice were then stimulated with these culture supernatants alone and pDC survival was determined (**Figures 4B, C**). Use of *Tmem173^–/–^* DCs that lack STING expression eliminated the possibility that residual ecto-nucleotide pyrophosphatase phosphodiesterase degradation resistant 3’3’-cGAMP/lyovec complexes (45) present in the supernatants, and/or STING ligands released from dead and dying cells, could activate STING directly in freshly isolated DCs (**Figure 4B**). Hence, if an inducible soluble mediator was indirectly inducing pDC death both WT C57BL/6 and *Tmem173^–/–^* pDCs should die. Importantly, we observed that pDCs from WT C57BL/6, but *not Tmem173^–/–^*, mice displayed significant cell death after stimulation with culture supernatants (**Figure 4C**), suggesting that pDC depletion after STING activation was not caused by a secreted soluble factor but rather *via* direct intracellular STING activation.

### Intrinsic Apoptosis Is Indispensable or cDC Death but Only Partly Required for pDC Death Induced Upon STING Activation

**Figure 5:**
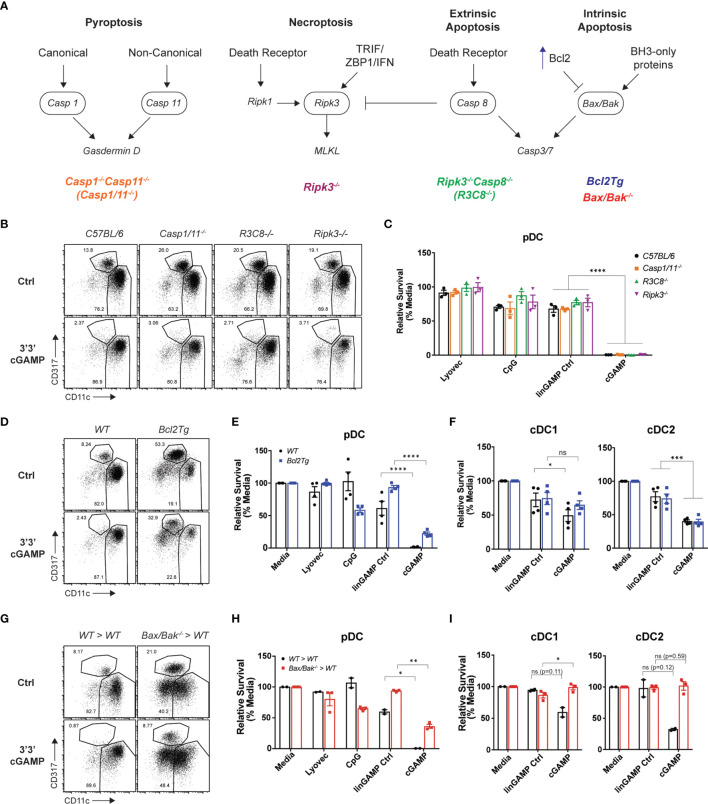
Blocking intrinsic apoptosis partially inhibits STING-dependent pDC death and rescues cDC death. **(A)** Schematic diagram showing death signaling pathways: encircled components highlight deleted genes in mice upstream of effector proteins gasdermin D, mixed lineage kinase domain-like (MLKL) protein and caspase-3 and caspase-7. **(B)** FACS plots or **(C)** bar graphs show mean relative survival ± SEM from 3 individual mice per genotype from C57BL/6, *Casp1/11^-/-^*, *Ripk3^-/-^Casp8^-/-^ (R3C8^-/-^)* and *Ripk3^-/-^* bulk splenic DCs stimulated with 10 nmol 3’3’ cGAMP or its linearized control ligand (linGAMP Ctrl) complexed with lyovec, lyovec alone or 0.5 μM CpG2216 for 18h. **(D)** FACS plots or **(E, F)** bar graphs from WT or *Bcl2Tg* bulk splenic DCs cultured with stimuli conditions as per **(B)**. Bar graph shows mean relative survival of **(E)** pDCs or **(F)** cDC subsets, mean ± SEM from 4 individual mice per genotype compiled from 2 independent experiments. **(G)** FACS plots and **(H, I)** bar graphs showing bulk splenic DCs from BM chimeras generated using Ly5.2 WT or *Vav-Cre Bax^-/-^/Bak^-/-^* mice cultured with stimuli conditions as per **(B)**. Bar graphs show Ly5.2^+^ relative survival of **(H)** pDCs or **(I)** cDC subsets, mean ± SEM from 2-3 individual mice per genotype. Statistical analysis was performed using **(C)** two-way ANOVA using Tukey’s test to correct for multiple comparisons or **(E, F, H, I)** two-tailed Paired Student’s *t* test where *P < 0.05, **P < 0.01, ***P < 0.001, ****P < 0.0001 and ns, not significant.

Having established that mouse STING-mediated pDC death is cell intrinsic, we next investigated which programmed cell death pathway was responsible. As multiple modes of cell death have been implicated in mammalian and bacterial/viral DNA sensing (46), as well as during STING responses in myeloid cells, fibroblasts and other cell types (47) we examined the role of four major inflammatory and non-inflammatory cell death pathways in pDC demise: pyroptosis, necroptosis, extrinsic and intrinsic apoptosis. A schematic diagram displaying key signaling components of each pathway is shown in **Figure 5A**. To determine which cell death pathway was involved in STING-dependent pDC death, we used genetically modified mice that had key components of each pathway deleted or overexpressed. *Caspase-1^-/-^Caspase-11^-/-^* (*Casp1/11^-/-^*) and *Ripk3^-/-^* mice were used to block pyroptosis and necroptosis, respectively. In addition to its key role in extrinsic apoptosis, caspase-8 can also cleave RIPK3 to limit necroptosis (48). Therefore, *Ripk3^-/-^Caspase-8^-/-^* (*Ripk3^-/-^Casp8^-/-^*) mice were also used to inhibit both extrinsic apoptosis and necroptosis simultaneously. We isolated DCs from these knockout mice and stimulated them with cGAMP for 18 hours. We observed pDC depletion of comparable severity to C57BL/6 mice in *Casp1/11^-/-^*, *Ripk3^-/-^* and *Ripk3^-/-^Casp8^-/-^* mice after cGAMP stimulation, thus eliminating the involvement of pyroptosis, necroptosis and extrinsic apoptosis, respectively, in STING-mediated pDC death (**Figures 5B, C**).

Next, to determine any involvement of intrinsic “mitochondrial BCL-2 family regulated” apoptosis in STING-dependent pDC death, we used Bcl2 transgenic mice that overexpress anti-apoptotic protein BCL-2 under the haematopoietic-restricted Vav promoter (*Bcl2Tg*). Importantly, BCL-2 has previously been shown to be a key baseline pro-survival factor in pDCs but not cDCs (49, 50). We isolated splenic DCs from WT non-transgenic or *Bcl2Tg* mice and stimulated them with 3’3’ cGAMP *in vitro*. Consistent with our previous findings (49, 50), there was a greater percentage of pDCs in the *Bcl2Tg* spleens (**Figure 5D**). Moreover, whilst we recovered more pDCs back from cultures of *Bcl2Tg* pDC treated with cGAMP, there was still a 72% decrease in pDC number compared to stimulation with its control ligand, suggesting that the major mechanism of pDC death post-STING activation is not rescued by BCL-2 overexpression (**Figure 5E**). Likewise, the cell death seen in STING-activated cDC populations was also not rescued by BCL-2 overexpression (**Figure 5F**).

Although BCL-2 is a crucial regulator of pDC survival, or at least its transgenic expression is able to support pDC survival (49), there are other anti- and pro-apoptotic signals (e.g. BH3-only proteins) that can influence the activation of intrinsic apoptosis. Hence, to more definitively determine if intrinsic apoptosis is involved in STING-dependent pDC death, we examined STING-mediated cell death in mice lacking the intrinsic apoptosis effector proteins, BAX and BAK. BAX and BAK activation and pore formation in the mitochondrial outer membrane is the ‘point of no return’ in apoptosis (recently reviewed in (51, 52)). Mice harbouring global genetic deletion of both *Bax* and *Bak* genes are embryonically lethal and therefore viable *Vav^cre^Bax^lox/lox^Bak^–/–^* mice were used to generate bone marrow (BM) chimeras lacking BAX and BAK specifically in haematopoietic cells (53). As per prior experiments, we isolated splenic DCs from these mice and stimulated them with 3’3’ cGAMP. The BM chimera splenic DC responded to cGAMP stimulation, in fact trending higher in the production of IFN-λ, IFN-α and TNF than the WT chimeras (**Supplementary Figure 9A**). As expected, the pDC population was depleted in the WT BM chimera spleen cultures after cGAMP stimulation, illustrating that STING-dependent pDC death still occurred in these BM chimeric mice (**Figure 5G**). However, in line with our *Bcl2Tg* results, pDCs from the *Bax^-/-^/Bak^-/-^* BM chimeras were partially rescued from cell death after cGAMP stimulation (**Figure 5H**), with survival increased about 40% in pDC deficient in expression of BAX and BAK. The trend of increased IFN-α from the STING-activated BAX/BAK deficient DC cultures (**Supplementary Figure 9A**) is likely due to the increased survival of these cells. Indeed, the *Bax^-/-^/Bak^-/-^* pDC also showed clear STING-dependent activation with upregulation of both CD69 and CD86 on these increased numbers of surviving cells (**Supplementary Figure 9B**). These data are in line with publicly available RNA sequencing data indicating normal levels of STING pathway genes (e.g., *Tmem173*, *Tbk1*, *Irf3*) in immune cells deficient in expression of BAX and BAK (54). These results confirm a partial contribution of intrinsic apoptosis to STING-dependent pDC death. The rigour of this finding was highlighted by the fact that, in contrast to the partial rescue of mouse pDC death observed in *Bax^-/-^/Bak^-/-^* BM chimeras after cGAMP stimulation, a complete rescue of the moderate levels of cell death in both cDC1 and cDC2 was observed, identifying that intrinsic apoptosis is the main mechanism of STING-dependent death of cDC (**Figure 5I**). These data further demonstrate that, similar to our findings relating to differential IFN production from cDCs and pDCs, it appears these subsets may elicit alternative (at least in part) modes of cell death.

To further substantiate our findings showing intrinsic apoptosis was involved in the rapid STING-induced death of pDC, we first treated DC with the pan-caspase inhibitor Q-VD-OPh (55). The pDC were rescued from cell death induced in just media alone or STING-induced (**Supplementary Figure 9C**). The cleavage of caspase 3 occurs downstream of BAX/BAK activation and at 1.5 h we show that caspase 3, although expressed at low levels, is indeed cleaved in pDC (**Supplementary Figure 9D**). Moreover, caspase 3/7 activity in the cGAMP-stimulated pDC was illustrated using the caspase 3/7 Glo-assay (Promega, **Supplementary Figure 9E**).

### Human DCs Display Divergent Responses to STING Activation

Our data thus far indicated that STING activation in mouse DC subsets induces DC activation and differential type I IFN production between subsets, and IFN-λ production from all subsets examined. cDC1 and cDC2 were also susceptible to STING-driven cell death and this was completely dependent on intrinsic apoptosis. We therefore turned to the human immune system to determine whether human DC subsets displayed similar phenotypes. Interrogation of publicly available microarray and RNA sequencing data sets revealed that, similar to mouse DC subsets, human cDC1 (CD141^+^) expressed the lowest levels of STING transcripts (**Supplementary Figure 3B–E**). The human cDC2 (CD1c^+^) expressed the highest level of STING amongst the DC subsets of human blood, with blood pDCs displaying intermediate STING expression between cDC1 and cDC2 subsets (**Supplementary Figure 3B**). Expression of STING transcripts in humanised mice DC subsets was similar, although pDC tended to have expression levels closer to cDC2 (**Supplementary Figure 3B–E**).

**Figure 6:**
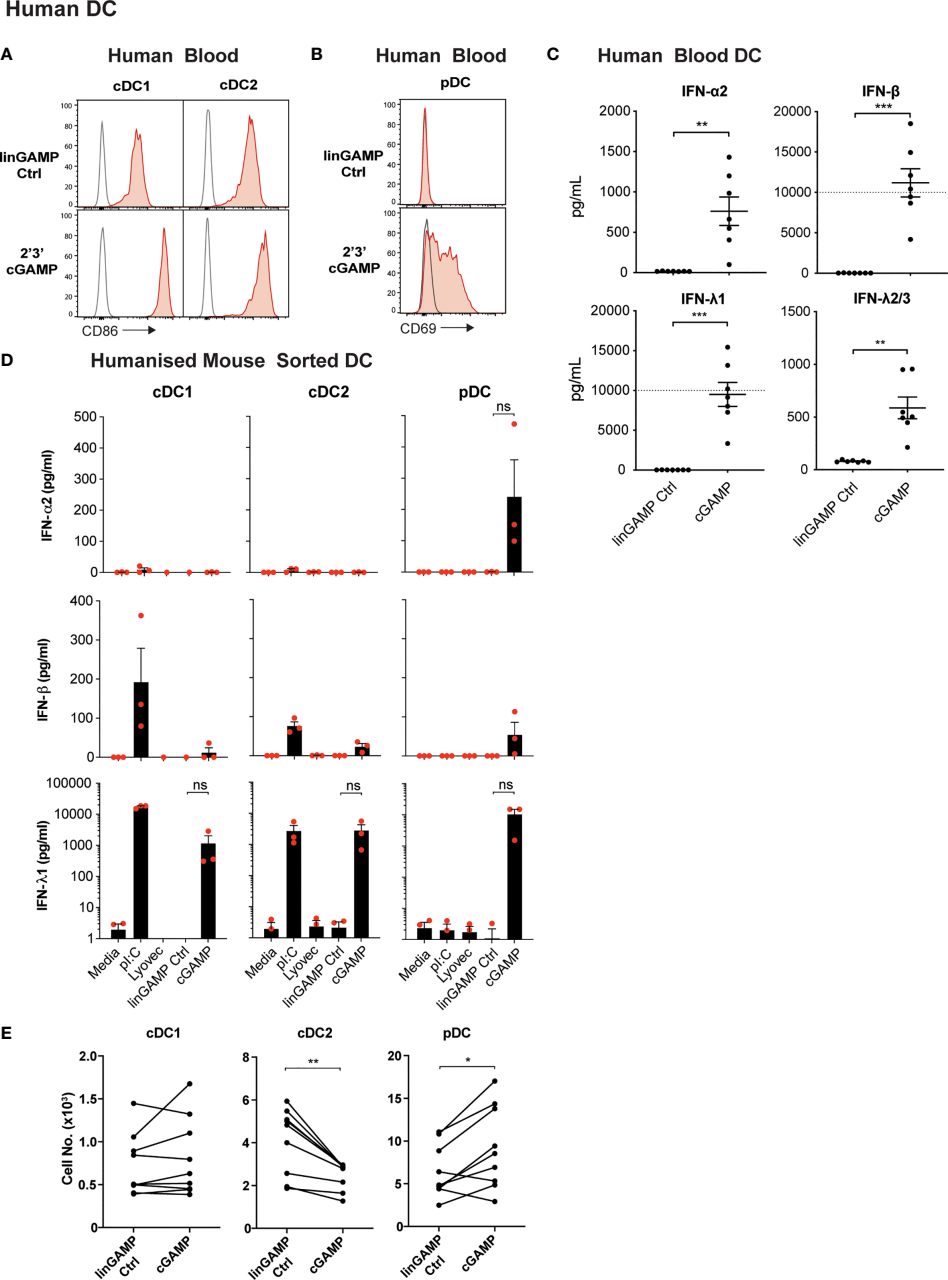
Human DCs demonstrate potent activation and differential IFN production, but not pDC death, after cGAMP stimulation. **(A–C)** Human blood DCs were stimulated with 5 nmol 2’3’ cGAMP or its linearized control ligand (linGAMP Ctrl) complexed with lyovec for 18 hrs. Histograms show activation marker expression on human blood **(A)** cDCs or **(B)** pDCs. Grey unfilled lines represent isotype control and red filled lines represent Ab stain. **(C)** Dots represent IFN production by DCs from each donor and lines show mean ± SEM of 7 donors from 3 independent experiments. Dotted line represents upper limit of quantification. **(D)** Sorted humanised mice DCs from BM were stimulated with 10 μg/mL pI:C, 10 nmol 2’3’ cGAMP or its linearized control ligand (linGAMP Ctrl) complexed with lyovec or lyovec alone for 18h. Bar graphs depict IFN production by each humanised mice DC subset showing mean ± SEM from 3 independent experiments. Human DC IFN production was analysed by flow cytometric bead assay. **(E)** Human blood DCs were stimulated with 5 nmol 2’3’ cGAMP or its linearized control ligand complexed with lyovec for 18 h and DC numbers enumerated by flow cytometry. Line graphs show paired individual cell numbers for each donor for each DC subset (n=9). Statistical analyses were performed using two-tailed Paired Student’s *t* test where *P < 0.05, **P < 0.01, ***P < 0.001 and ns, not significant.

Recently, Pratik et al. (27) demonstrated that human pDC, in line with their mouse counterparts, also produced type I IFN and IFN-λ, specifically IFN-λ1, upon STING activation. To determine whether STING stimulation with cGAMP also induces potent activation of *all* human DCs, cDC and pDC populations were isolated from human blood (**Supplementary Figure 4B**) and were stimulated with 2’3’ cGAMP, which has been reported to be the most stimulatory STING ligand for human cells (56). Upon cGAMP stimulation the levels of activation marker CD86 on cDCs (**Figure 6A**) and CD69 on pDCs were upregulated (**Figure 6B**). Examination of DCs from humanised mice, previously shown to be functionally akin to their human blood counterparts (57), also demonstrated a potent increase in CD80 expression by pDCs and cDC subsets (**Supplementary Figure 10A**). Therefore, as we observed in mouse DC subsets (**Figure 1**), 2’3’ cGAMP is a strong activator of all human DC subsets.

To investigate whether human DCs similarly responded with IFN production to STING ligands, we isolated total DCs from human blood and stimulated them with 2’3’ cGAMP and controls and measured IFN production. Like mouse splenic DCs, human DCs produced IFN-α, IFN-β and IFN-λ2/3 in response to the cGAMP (**Figure 6C**). However, IFN-λ1 (IL-29), an isotype of IFN-λ that is a pseudogene in the mouse genome, was produced at extremely high levels in response to cGAMP (**Figure 6C**). As with mouse DCs (**Figure 1**), we observed human blood DC IFN-β responses were also much greater than IFN-α responses to STING ligand (**Figure 6C**).

We have previously shown that the activation of humanised mice DC subsets closely mirrors that of *ex vivo* blood DC of human donors (57–61), and using these mice is far more practical to obtain enough human DC numbers suitable for analyses of individual subset function. Therefore, to determine the specific human DC subsets responsible for IFN production in response to STING ligand, we examined 2’3’ cGAMP-induced IFN production in sorted DC subsets from humanised mice. pDC were the only subset found to produce detectable levels of IFN-α2, whilst all subsets produced low levels of IFN-β in response to 2’3’cGAMP (**Figure 6D**). Strikingly, all humanised mice DC subsets also produced large amounts of IFN-λ1 after stimulation with 2’3’cGAMP (**Figure 6D**), although this was found to be statistically not significant due to human cord blood derived-DC donor-to-donor variation in STING responses. This variation in the absolute amount of cytokines produced by human DC in response to STING activation was also observed in the enriched blood DC responses (**Figure 6C**). In most experiments, the production of IFN-λ2/3 was not detected above background in response to cGAMP stimulation of the sorted humanised mice DC subsets (not shown). Thus, our data reveal for the first time that *all* mouse and human DC subsets produce IFN-λ in response to ligands of the cytoplasmic CDN sensor, STING. Of note, we have discovered that the human restricted IFN-λ1 is the IFN produced most highly by *all* human DC subsets.

### Human pDCs Are Resistant to STING-Induced Cell Death

We uncovered that STING activation causes rapid death of mouse pDCs that was partially dependent on intrinsic apoptosis. Intriguingly, enumeration of DC subsets from human blood DC cultures or isolated DCs from humanised mice after 18 hours stimulation with 2’3’ cGAMP or its linearized control, indicated that the obliteration observed for murine pDCs upon STING activation was *not* translated in the human system (**Figure 6E** and **Supplementary Figure 10B**). In fact, pDC death was actually rescued by activation with 2’3’ cGAMP in most human donors (**Figure 6E**). The CD141^+^ cDC1 subset showed little survival difference between the control and 2’3’ cGAMP stimulation conditions, with minor variations between donors (**Figure 6E** and **Supplementary Figure 10B**). However, the blood cDC2 subset exhibited significantly enhanced cell death in the presence of 2’3’ cGAMP in all donors tested (**Figure 6E**). Interestingly, the observed death of human blood cDC2 was not conserved in the cDC2 of humanised mice that were similarly challenged with 2’3’ cGAMP (**Supplementary Figure 10B**). Thus, our data reveal strong species differences between DC responses to STING activation, with heightened cell death exhibited by mouse but not human pDCs.

An open question here was whether the IFN-λ1, only produced by the human DC, could have been protecting the human pDC from STING-induced death. However, experiments which stimulated human DC with STING ligands in the presence of IFN-λ1 neutralising antibodies, did not lead to enhanced cell death of human pDC (**Supplementary Figure 10C**). These results suggested that survival of human pDC after STING activation was independent of their production of IFN-λ1. However, the IFN-λ1 neutralising antibodies did abrogate the upregulation of CD69 on the human pDC, indicating that the upregulation of this ISG is dependent on active IFN-λ signaling in the cultures (**Supplementary Figure 10D**).

## Discussion

A protective role of IFN-λ in protecting against mucosal anti-viral responses is clear. Potential roles of IFN-λ in many other diseases have been proposed by GWAS studies revealing associations with polymorphisms in the IFN-λ locus. In this study, we show for the first time that all major subsets of mouse and human DC, key professional antigen presenting cells linking innate and adaptive immunity, produce IFN-λ in response to direct STING activation. STING signaling is implicated in many different diseases, raising the possibility that IFN-λ production from DC underpins at least some phenotypes of STING-mediated disease.

IFN-λ2/3 are the only IFN-λs expressed in mouse and mouse cDC1 were the prominent producers in response to STING activation (**Figure 2D**), although cDC2 and pDC also produced low levels. In the human DC, where IFN-λ1, 2 and 3 genes are present, IFN-λ1 was by far the *predominant* IFN produced in response to STING ligands (**Figures 6C, D**). Strikingly, we were also able to show that distinct type I IFNs are produced by human and mouse DCs to STING ligands. Namely, IFN-α is produced at low levels by all mouse DC but solely by human pDCs in response to STING activation (**Figures 2D**, **6D**), while amongst the mouse DC, only mouse cDC2 produce IFN-β (**Figure 2D**) and all human DC subsets produce low levels of IFN-β (**Figure 6D**).

We have provided evidence that STING-mediated signaling, at least in the mouse DC, differs between cDC and pDC subsets. The signaling downstream of STING activation in the mouse DC subsets included the characteristic TBK1 phosphorylation, STING phosphorylation and IRF3 activation in all subsets (**Figure 2A**). However, surprisingly, STING-mediated NF-κB activation (read out as phosphorylation of the NF-κB p65 subunit) and associated cytokine production was very weak in the STING competent pDC subset despite these cells expressing the highest levels of total STING protein. Of note, this difference in STING activation was concomitant with rapid death of the mouse pDC (significant by 4 hours after stimulation), involving intrinsic apoptosis. We have previously found differential reliance on NF-κB subunits for pDC activation and cytokine production compared to cDC (49). This, together with the high basal IRF7 expression by pDC (62) may explain the lack of NF-κB p65 activation, but requires further investigation.

The human IFN-λ response to STING activation was unusual in that it was predominated by IFN-λ1. Depending on the donor, 10–30-fold more IFN-λ than IFN-λ2/3 was produced by human DC in response to STING activation (**Figure 6C**). We have not previously seen this predominant IFN-λ1 production when examining human DC responses to TLR3 stimulation, where IFN-λ1 and IFN-λ2/3 were produced at similarly high levels (26). IFN-λ1 production by cDC1, proposed to be *via* TLR3, has recently been associated with positive clinical outcomes and local Th1 immune responses in breast cancer patients (63). In these patients, IFN-λ1 transcripts were produced at similar levels to the combined IFN-λ2/3 transcripts, aligning with what we have previously seen at the protein level with poly I:C stimulation in human DCs (26). The downstream immune response elicited by STING activation of DCs, with a predominance of IFN-λ1 over IFN-λ2/3 production by *all* DC subsets is not yet clear. Previous work indicated a potential lack of gene repressor function by IFN-λ1 compared with IFN-λ2 on epithelial cells (33). Our findings certainly warrant future examination of the effects of IFN-λ1 production in the context of STING activation.

The role of the cytosolic CDN sensor STING in anti-tumour immunity has been attributed to its recognition of tumour DNA within cDC1s, which consequently triggers type I IFN production and enhances DC cross-priming of tumour antigen to cytotoxic T cells (24). As a result, STING agonists have been promoted as potent adjuvants in cancer immunotherapies. However, not only does the cDC1 subset have the lowest expression of STING amongst the DC subsets (**Supplementary Figure 2**), they also do not produce high quantities of IFN-I after STING activation (**Figures 2D**, **6D**). Our results suggest that cDC2 and pDCs could contribute to IFN-I production in the anti-tumour cytokine milieu, which has been previously postulated (64). In contrast to the well documented role for STING-induced type I IFNs in anti-tumour immunity, the role of IFN-λ has not been investigated up until this point. We have previously shown that TLR3-induced IFN-λ production is blunted in DCs lacking the Type I IFN receptor (IFNAR) (26), thus studies showing a requirement for IFNAR signaling in the efficacy of STING responses could also be masking the involvement of downstream IFN-λ signaling. An anti-tumour role for IFN-λ is an attractive idea, as IFN-λR is expressed on neutrophils, DCs, throughout the mucosal epithelia, within epithelial cells of the liver, kidney and brain, as well as on various tumours (31, 65). Moreover, IFN-λ treatment has been shown to synergise with the kinase inhibitor sorafenib to inhibit Hepatocellular carcinoma cell growth and induce apoptosis (66), and a strong correlation between IFN-λ1, 2, and 3 producing cDC1 and beneficial outcomes in breast cancer has recently been described (63). Therefore, the fact a large amount of IFN-λ is produced by DCs in response to STING activation warrants further investigation in the realms of cancer immunotherapy. However, as the IFN-λ locus has been shown to be polymorphic in a subset of cancer patients and patients suffering from infectious diseases (8, 29, 65, 66), moving forward, polymorphisms and differences in IFN-λ gene expression will need to be taken into account when considering clinical STING immunotherapeutic approaches (29, 35, 67, 68).

It is of interest that some studies have implicated STING-induced IL-10 as having an inhibitory effect on anti-tumour immunity (69, 70) and in some cases, a protective effect to avoid continued inflammation and development of colorectal cancer (43). Our studies show that DCs do not produce IL-10 in response to cGAMP stimulation but it is worth noting that the IFN-λ1 receptor shares with the IL-10 family the use of IL-10 R2. Whether the extremely high levels of IFN-λ1 expressed by human DC in response to STING stimulation could lead to a usurping of the IL-10R2 to the IFN-λR complex, and thus result in a decrease of available IL10R2 for IL-10 signaling, remains to be elucidated.

Importantly, mouse and human pDC survival in response to STING activation was divergent, with human pDC showing equivalent or enhanced survival *in vitro* to STING activation (**Figure 6E** and **Supplementary Figure 10B, C**), whereas mouse pDC were rapidly and potently killed *in vitro* (**Figure 3**). This death was STING-dependent (**Figure 3D**) and involved the intrinsic apoptotic pathway, although it was not the sole mechanism for STING-induced killing of pDCs (**Figure 5** and **Supplementary Figure 9**). This finding contradicts the pathways reported to mediate STING-dependent cell death observed in malignant lymphocytes and other myeloid cell types (71–77). Lysosomal rupture or other forms of phagocytic cell death may be compensatory mechanisms, given the co-localisation between STING and autophagy-related proteins (10). Alternatively, the rapid death of the murine pDC may be a novel form of cell lysis. A further cell death anomaly we observed was that both mouse splenic (**Figure 3B**) and human blood (**Figure 6E**) cDC2 but not humanised BM cDC2 were sensitised to STING-mediated intrinsic apoptotic cell death (**Figure 5I** and **Supplementary Figure 10B**). The lack of death in the humanised mice cDC2 of BM origin suggests that the non-haematopoietic niche may contribute to priming of these cells for STING-mediated death. Whether this is mediated by soluble factors or cell-mediated interactions remains to be elucidated.

Divergent outcomes in DC viability and high production of IFN-λ1 after STING activation mark major points of difference between species and need to be considered when translating experimental results from mouse models to human clinical trials. Of particular importance, pDC in the tumour environment are often associated with poor prognosis (78–81). Our data would suggest that in mouse tumour models, exposure to STING ligand adjuvants would eliminate pDCs. In humans treated with STING adjuvants, activated pDCs would potentially persist in a tumour environment. Hubert et al. (63) recently demonstrated a correlation between pDC infiltration in breast tumours and Treg accumulation. This suggests that pDCs activated by STING could similarly lead to an immunosuppressive microenvironment, although this remains to be elucidated. We are yet to define what the key mediators directing differental STING signaling outcomes between mouse and human pDCs are, however elucidating these molecular differences could lend insight into discrete mechanisms of inducing rapid pDC death, tools that could potentially be harnessed to improve cancer immunotherapies or alter viral immune responses.

In summary, this work places IFN-λ in the spotlight as a potential major player in DC-mediated immune responses downstream of STING activation. It also highlights the need to re-evaluate STING responses in mice, in particular, does the direct killing of murine pDC have beneficial anti-tumour effects, or indeed anti-viral effects, that are not recapitulated in the human setting?

## Methods

### Mice

All mice were housed under specific pathogen free conditions and all animal experimental procedures were approved by either Monash University or Walter and Eliza Hall Institute (WEHI) animal ethics committees. C57BL/6 (WT) mice were obtained from either Monash Animal Research Platform or WEHI and all transgenic mice were obtained from WEHI. *Tmem173^-/-^* mice (9) were provided by Benjamin Kile (Biomedicine Discovery Institute, Monash University, Australia). *Vav-Bcl-2* transgenic mice (*Bcl2Tg*) (82) were provided by Yifan Zhan (WEHI, Australia). *Ripk3^-/-^, Ripk3^-/-^Casp8^-/-^* mice (83) and *Vav-Cre Bax^lox/lox^Bak^-/-^* (*Bax^-/-^/Bak^-/-^*) BM chimeras (84) were provided by Kate Lawlor (Hudson Institute of Medical Research, Australia) and James Vince (WEHI). *Casp1^-/-^Casp11^-/-^* mice (85) were provided by Seth Masters (WEHI, Australia). Mice used were 6-12 weeks old except for *Bax^-/-^/Bak^-/-^* BM chimeric mice that were 10 months old.

### Mouse DC Purification

DCs from spleen were isolated using a previously described method (86). In brief, spleens were digested using DNase/Collagenase (Roche Diagnostics, Basel, Switzerland/Worthington Biochemical Corporation, Lakewood, New Jersey) at room temperature (RT) for 20 min and filtered to create a single cell suspension. Light density cells were isolated by centrifugation in a 1.077 g/cm^3^ NycoPrep™ medium (AXIS Shield PoC AS, Dundee, Scotland). Negative selection using a rat monoclonal Ab cocktail against CD3-ϵ (KT3-1.1), Thy-1 (T24/31.7), Ly6G/Ly6C (1A8), CD19 (ID3) and erythrocytes (TER-119) together with anti-rat immunoglobulin immunomagnetic beads (Qiagen, Hilden, Germany) was used to isolate DCs. For sorted DCs, these isolated cells were then labelled with the following fluorophore-conjugated mAbs: CD11c (N418), CD45RA (14.8), CD8 (53-6.7), CD11b (M1/70), CD49b (DX5) (Becton Dickinson, Franklin Lakes, New Jersey; eBioscience, San Diego, California; BioLegend, San Diego, California; TONBO, San Diego, California) and sorted on BD Influx machine according to gating strategy provided (**Supplementary Figure 4A**).

### Human Blood DC Purification

Human blood (15-20 mL) collected in heparin tubes was diluted with PBS (human osmolarity) and underlayed with 10 mL Ficoll-Hypaque 1.077 g/mL density medium (Thermofisher Scientific, Waltham, Massachusetts) at RT. Samples were then centrifuged at 400 g for 30 min at 20°C with the brake off. The peripheral blood mononuclear cell (PBMC) layer was then collected, washed twice and counted. DCs were negatively selected from PBMCs using EasySep™ Human Pan-DC pre-enrichment kit (STEMCELL™ Technologies, Tullamarine, Victoria) according to manufacturer’s instructions.

### Humanized Mouse DC Purification

Human CD141^+^ DC, CD1c^+^ DCs and pDCs were isolated from the bone marrow and spleens of humanised mice as described previously (57). Briefly, humanised mice were generated by engrafting immunodeficient neonatal NSG mice with human cord blood CD34^+^ haematopoietic progenitor cells (HSC), leading to multi-lineage human immune reconstitution, including functional human DC subsets, from 12 weeks of age (57). Cord blood was obtained from the Queensland Cord Blood Bank following written informed consent in accordance with Mater Adult Hospital Human Ethics Committee approval. Mice were housed and treated in accordance with approval by the University of Queensland Animal Ethics Committee and under TRI Biological Resources Facility (BRF) operations. Following immune reconstitution, DC populations were expanded with 2 *s.c.* injections with 50 μg Flt3L (Bio-X Cell, Lebanon, New Hampshire) 4 days apart. Where indicated, gene expression data were obtained from humanised DC that were activated *in vivo* by administration of HBSS (control) 50 μg high molecular weight poly I:C (*In vivo*Gen), 20 μg R848 (*In vivo*Gen), or poly I:C and R848 combined, as previously described (57).

Humanised mouse DC were enriched by magnetic bead depletion of mouse CD45, using rat anti-mouse CD45 (Beckman Coulter, Pasadena, California, 30-F11) and TER-119 and rat anti-human CD14 (Beckman Coulter, RMO52), CD19 (Beckman Coulter, J3-119), CD3 (Scientific support, OKT3) and CD34 (Beckton Dickson, MY10) Abs and rat anti-mouse TER-119 (BioLegend, TER-119) followed by sheep anti-rat IgG Dynabeads (Thermofisher Scientific) separation. Obtained single cell suspension was then stained for flow cytometry sorting using: LIVE/DEAD Fixable Aqua (Thermofisher Scientific), huCD45 APC Cy7 (BioLegend), Lineage (CD3, CD14, CD16, CD19, CD20 and CD56) PacBlue (BioLegend), HLA-DR PE-Cy7 (BioLegend), CD123 PerCP5.5 (BioLegend), CD141 APC (BioLegend), CD1c PE (BioLegend). Human DCs were gated on a live cell gate as huCD45^+^huHLA^-^DR^+^Lineage^-^. cDC1 were further defined as CD141^+^CD1c^-^, cDC2 as CD141^-^CD1c^+^ and pDC as CD141^-^CD1c^-^CD123^+^ (**Supplementary Figure 4B**).

### *In Vitro* Stimulations

Mouse spleen and human blood DCs were resuspended in RPMI-1640 Glutamax (Thermofisher Scientific) with 10% FCS (*In vitro* Technologies™, Noble Park, Victoria), 100μM 2-Mercaptoethanol (Thermofisher Scientific) and 0.01% Penicillin/Streptomycin (Thermofisher Scientific) and humanized mice DCs were resuspended in RPMI containing 10% FCS, 10 mM HEPES, 1mM sodium pyruvate, penicillin, streptomycin, 2 mM L glutamine, NEAA and 50 μM β-mercaptoethanol and were plated at a concentration of 0.5x10^6^/mL in 96-well round bottom plates. They were stimulated with 0.5 or 10 μM cytosine-phosphate guanosine (CpG) oligonucleotides 2216 or 1668 (Geneworks, Thebarton, South Australia; Miltenyi Biotech, Bergisch Gladbach, Germany), 10-100 μg/mL high molecular weight polyinosinic:polycytidylic acid (poly I:C) (*In vivo*Gen) or 10 nmol transfected 3’3’ cGAMP (c[G(3’,5’)pA(3’,5’)p]), 2’3’ cGAMP (c[G(2’,5’)pA(3’,5’)p]), c-di-AMP, c-di-GMP (*In vivo*Gen) or their respective linearized control ligands 3’5’-pGpA, 2’5’-GpAp, 5’-pApA and 5’-pGpG (*In vivo*Gen) at 37°C, 10% CO_2_ for 18 hrs unless indicated otherwise. The following reagents were included in some experimental cultures as indicated in the figure legends: 0.2ng/mL recombinant mouse GM-CSF (Peprotech, East Windsor, New Jersey), 5μM Q-VD-Oph (Selleck Chemicals, Houston, Texas), 30ng/mL recombinant human IL-29 (R&D Systems, Minneapolis, Minnesota), 5μg/mL anti-human IL-29 (neutralising Clone MAB15981, R&D systems).

### CDN Transfection

Cyclic dinucleotides (CDNs) were incubated with Lyovec (*In vivo*Gen) at RT for 15min before being used for stimulations.

### Flow Cytometry

Single cell suspensions were incubated with Fc Block (FcγRIII/II, 2.4G2) for 10 min before labelling with the following anti-mouse fluorophore-conjugated mAbs (BD, eBiosciences, BioLegend, TONBO, in house): CD11c (N418), CD11b (M1/70), CD317 (120.G8 or 927), CD8 (53-6.7), CCR9 (CW-1.2), CD3-ϵ (17A2), CD49b (DX5), CD86 (GL1), CD80 (16-10A1) and MHCII (M5/114.15.2) or anti-human fluorophore-conjugated mAbs (BD, eBiosciences, BioLegend, Miltenyi, in house): CD1c (L161), CD3-ϵ (BC3), CD11c (B-ly6), CD14 (FMC17), CD16 (3G8), CD19 (FMC63), CD20 (B1), CD34 (AC133), CD57 (HNK1.1), CD69 (FN50), CD86 (IT2.2), CD123 (7G3), CD141 (AD5-14H12), Glycophorin A (10F7MN) and HLA-DR (REA332). Sorted humanised mouse DCs were labelled with FITC-conjugated CD80 (BioLegend). Dead cells were excluded using propidium iodide (Merck Millipore, Darmstadt, Germany) or LIVE/DEAD Fixable Aqua (Thermofisher Scientific) dyes and samples were acquired on either Fortessa or LSRII flow cytometer (BD). Sphero or TruCOUNT beads (BD) were used to determine cell counts for human DCs. Analysis was conducted using FlowJo software (Tree Star, Ashland, Oregon).

### IFN ELISAs

Murine IFN-λ and IFN-α in supernatants were assayed using sandwich ELISA mAbs from RnD Systems, Minneapolis, Minnesota (IFN-λ) or *In vivo*Gen (IFN-α). Immunosorbent plates (Thermofisher Scientific) were coated with 1 μg/mL rat IgG2b anti-mouse IL-28a/b or 2 μg/mL anti-mouse IFN-α capture mAbs at 4°C overnight in a humidified box. Plates were washed between each step using 0.05% Tween (Sigma-Aldrich, St Louis, Missouri)/PBS and 1% bovine serum albumin (Sigma-Aldrich)/PBS was used for blocking at RT for 1 hr. Supernatants were added to plates and incubated at 4°C overnight. Recombinant mouse IL-28a/b (Cat No: 1789-ML-025) or IFN-α (Cat No: re-mifna) were used as standards. 0.25 μg/mL rat IgG2b anti-mouse IL-28a/b or 30 ng/mL anti-mouse IFN-α biotinylated detection mAbs were added to plates and incubated for 2 hr at room temperature. Streptavidin-horseradish peroxidase (GE Healthcare, Chicago, Illinois) was used to develop ELISA substrate containing 0.1 M citric acid, 548 μg/mL 2,2′-azino-bis(3-ethylbenz-thiazoline-6- sulfonic acid (ABTS), Sigma-Aldrich) and H_2_O_2_. ELISA results were read at 405-490 nm wavelength reduction.

### Cytokine Analysis

All cytokines and chemokines, except for murine IFN-λ and IFN-α, in supernatants were analysed using flow cytometric bead-based assay LEGENDplex™ kit (BioLegend) according to manufacturer’s instructions and software.

### RNA-Sequencing and Bioinformatics Analysis

RNA from sorted mouse splenic DC subsets was isolated using an RNeasy Mini Kit (Qiagen) and residual genomic DNA removed using RNase-free DNase (Qiagen). Sample libraries were constructed using Illumina TruSeq Stranded mRNA kit according to manufacturer’s instructions. Libraries were quantitated using Qubit DNA HS kit (Thermofisher Scientific) and checked for adaptor contamination using Bioanalyzer 2100 (Agilent Technologies, Santa Clara, California). Sample libraries were then sequenced by Micromon on NextSeq500 using High-Output SBS chemistry (Illumina, San Diego, California) at 1.8pM library concentration and 1x75b read length. RNA-seq data analysis was performed in Degust (http://degust.erc.monash.edu/; version 4.1.1) by the Monash Bioinformatics Platform personnel, D.R Powell and A. Barugahare.

### Quantitative RT-PCR

Total RNA was extracted from sorted DC subsets and cDNA synthesized using SuperScript^®^ IV First-Strand Synthesis System (Thermofisher Scientific) according to the manufacturer’s protocol. The expression of genes was determined using quantitative real-time PCR. STING (*TMEM173*) and cGAS (*MB21D1*) qPCR Primers were purchased from QIAGEN. Each cDNA sample was amplified using SYBR Green Master Mix (Rox) (Thermofisher Scientific) on the Real-time PCR Viaa 7 system using cycles conditions as: 95˚C for 10 min, 45–50 cycles at 95˚C for 15 sec, and 60˚C for 60 sec. Reactions were run in triplicates for four independent experiments. The Ct (cycle threshold) values were normalized to the geometric mean of GAPDH as the housekeeping gene to control the variability in expression levels and were analyzed using the 2 ^-ΔΔCT^ method (87).

### Western Blotting

Western blotting of sorted DC lysates was performed according to a recently published protocol (13). The primary and secondary antibodies used in this assay were: Rabbit monoclonal anti-STING (D2P2F) (Cell Signaling Technology, Danvers, Massachusetts; Cat#13647), rabbit monoclonal anti-P-STING Ser^365^ (D8F4W) antibody (Cell Signaling Technology, Cat#72971), rabbit polyclonal anti-TBK1 antibody (Cell Signaling Technology, Cat#3013), rabbit monoclonal anti-TBK1 Ser^172^ (D52C2) antibody (Cell Signaling Technology, Cat#5483), rabbit monoclonal anti-IRF3 (D83B9) antibody (Cell Signaling Technology, Cat#4302), rabbit monoclonal anti-P-IRF3 Ser^396^ (4D4G) (Cell Signaling Technology, Cat#4947), rabbit monoclonal anti-NF-kappa-B p65 (C22B4) antibody (Cell Signaling Technology, Cat#4764), rabbit monoclonal anti-NF-kappa-B P-p65 Ser^536^ (93H1) antibody (Cell Signaling Technology, Cat#3033), rabbit polyclonal anti-cleaved caspase 3 (Asp175) antibody (Cell Signaling Technology, Cat#9661), mouse monoclonal anti-beta ACTIN, HRP (AC-15) (Abcam, Cambridge, Massachusetts; Cat#Ab49900) and Peroxidase-AffiniPure Goat Anti-Rabbit IgG (H+L) secondary antibody (Jackson ImmunoResearch Labs, West Grove, Pennsylvania, Cat#111-035-003).

### Caspase3/7 Detection Assay

Caspase3/7 activity was measured using Caspase-Glo®3/7 Assay (Promega, Madison, WI) according to manufacturer’s instructions.

### Statistical Analysis

Paired and unpaired two-tailed Student’s *t* tests, one- or two-way ANOVA was performed using Prism 7.0 (GraphPad software) where *P < 0.05, **P < 0.01, ***P < 0.001 and ****P < 0.0001.

## Data Availability Statement

The datasets presented in this study can be found in online repositories. The names of the repository/repositories and accession number(s) can be found below: GEO, GSE188992.

## Ethics Statement

The studies involving human participants were reviewed and approved by Monash University Human Research Ethics Committee Monash University, Clayton, Victoria, Australia. The patients/participants provided their written informed consent to participate in this study. The animal study was reviewed and approved by Monash Animal Ethics Committee MARP-2, Monash University, Clayton, Victoria, Australia.

## Author Contributions

ESP, GD, PT, AS, ZM, NJ, KB, and KAM performed the experiments. EP, GD, KB, PT, MB, KR, MW, and MO’K analyzed and interpreted results. EP, MO’K, HH, MW, CM, BK, DDN, and KL contributed to the experimental design. YZ, BK, and KL provided genetically modified mice. EP, GD, KR, and MO’K wrote the manuscript. All authors reviewed the manuscript. All authors contributed to the article and approved the submitted version.

## Funding

MO’K was supported by a National Health and Medical Research Council (NHMRC) Senior Research Fellowship (1077633), NHMRC Project grant (1085934) and Worldwide Cancer Research Grant (14-0281) and Worldwide Cancer Research / Cancer Australia Grant (20-0300). KR was supported by NHMRC project grants (1085934) and Mater Foundation grants; KL was funded by NHMRC project grants 1145788, 1162765 and 11801089 and is supported by an ARC Future Fellowship (FT190100266). DDN was supported by a ﻿Monash University FMNHS Senior Postdoctoral Fellowship. ESP was supported by a Monash University PhD scholarship and Worldwide Cancer Research / Cancer Australia Grant (20-0300) and GD was supported by a Griffith University PhD Scholarship. AS, MB, ZM were supported through Australian Government Research Training Program (RTP) scholarships. NJ was supported through a Monash University Faculty International Tuition Scholarship (FITS) and Co-funded Monash Graduate Scholarship (Co-MGS). KB was supported by a Monash Silver Jubilee Postgraduate Research scholarship (MSJS) and a Monash Graduate Excellence scholarship. The Translational Research Institute is supported by a grant from the Australian Government.

## Conflict of Interest

HH is an employee of Bavarian Nordic GmbH.

The remaining authors declare that the research was conducted in the absence of any commercial or financial relationships that could be construed as a potential conflict of interest.

## Publisher’s Note

All claims expressed in this article are solely those of the authors and do not necessarily represent those of their affiliated organizations, or those of the publisher, the editors and the reviewers. Any product that may be evaluated in this article, or claim that may be made by its manufacturer, is not guaranteed or endorsed by the publisher.

## Supplementary Material

Supplementary Figure 1 Mouse DCs are potently activated by CDNs and produce IFN-λ that is STING-dependent. **(A)** Bulk splenic mouse DCs were stimulated with 1 or 10nmol 2’3’ cGAMP, 3’3’ cGAMP, c-di-AMP or c-di-GMP complexed with lyovec, their respective linearized control ligands (Ctrl) complexed with lyovec, lyovec alone, 0.5 µM CpG2216 or 100 µg/mL pI:C for 18h. MHCII expression on DC subsets was determined using flow cytometry. Bar graphs represent the mean difference between geometric mean fluorescence intensities (gMFI) of stained samples and fluorescence minus one (FMO) controls ± SEM from 3 biological replicates (pool of 2 mice per replicate). **(B)** Bulk splenic mouse DCs from C57BL/6 or Tmem173-/- were stimulated with 10nmol 3’3’ cGAMP complexed with lyovec, lyovec alone or 0.5 µM CpG2216 for 18h. IFN-λ production in cell culture supernatants were analysed by ELISA. Bar graphs represent mean ± SEM from 3 individual mice per genotype. Statistical analyses were performed using two-tailed Paired Student’s t test where *P < 0.05, ** P < 0.01and ns, not significant.

Supplementary Figure 2 Mouse DCs upregulate activation markers CD86 and MHCII after CDN stimulation. Bulk splenic mouse DCs were stimulated with 1 or 10nmol 2’3’ cGAMP, 3’3’ cGAMP, c-di-AMP or c-di-GMP complexed with lyovec, their respective linearized control ligands (linGAMP Ctrl) complexed with lyovec, lyovec alone, 0.5 µM CpG2216 or 100 µg/mL pI:C for 18h. Histograms show CD86 and MHCII expression on DC determined using flow cytometry. Dotted lines represent fluorescence minus one (FMO) control and filled in lines represent Ab stain. Grey lines represent linGAMP Ctrl and red lines represent cGAMP samples. Histograms represent 1 of 3 biological replicates (pool of 2 mice per replicate).

Supplementary Figure 3 STING transcripts in mouse and human DCs. **(A)** *Tmem173* transcript expression (counts per million) from RNA-sequencing analysis of steady state sorted mouse splenic DCs. Bar graphs represent mean ± SEM from 3 independent samples of 10-14 mice pooled per sample. **(B)** Publicly available microarray data sets [www.stemformatics.org] represent expression levels of STING in human immune cells isolated from different organs (Heidkamp, 2016). **(C)** Microarray data demonstrating the changes in gene expression levels of STING in human cDCs when treated with polyI:C (TLR3 agonist), R848 (TLR7/8 agonist) and the combo (polyI:C+R848) (Minoda, 2017). **(D, E)** Quantitative PCR analysis was performed on immune cell subsets isolated from humanised mouse bone marrow to determine the pattern of STING expression in the steady state **(D)** as well as the expression levels after activation **(E)** in each DC subset. Gene expression levels were normalised to GAPDH. Each experiment was performed for 4 independent samples (n = 4) and the error bars represent mean ± SEM.

Supplementary Figure 4 Gating strategies for mouse and human DC subsets. **(A)** Bulk splenic DCs pooled from 15^+^17 C57BL/6 mice were labelled with the following fluorophore^+^conjugated mAbs: CD11c (N418), CD45RA (14.8), CD8 (53^+^6.7), CD11b (M1/70), CD49b (DX5). They were then sorted by first removing NK cells using junk gate and sorted into pDC (CD11c^int^CD317^hi^CD11b^-^), cDC1 (CD11c^hi^CD317^lo^CD8^+^CD11b^-^) and cDC2 (CD11c^hi^CD317^lo^CD8^-^CD11b^-^) subsets. **(B)** Purified human blood DCs were labelled with the following fluorophore^+^conjugated mAbs: CD1c (L161), CD3^-^ε (BC3), CD11c (B^+^ly6), CD14 (FMC17), CD16 (3G8), CD19 (FMC63), CD20 (B1), CD34 (AC133), CD57 (HNK1.1), CD69 (FN50), CD86 (IT2.2), CD123 (7G3), CD141 (AD5^+^14H12), Glycophorin A (10F7MN) and HLA^+^DR (REA332). DCs were first gated on HLA^-^DR^+^Lin^-^ cells followed by separation into cDC1 (CD11c^+^CD141^+^CD1c^-^CD123^-^), cDC2 (CD11c^+^CD1c^+^) and pDC (CD11c^-^CD123^+^).

Supplementary Figure 5 Sorted mouse DCs upregulate CD80 and CD86 expression after cGAMP stimulation. Sorted splenic mouse cDC1, cDC2 and pDCs from a pool of 15-17 mice were stimulated with 10 nmol 3’3’ cGAMP or its linearized control ligand (linGAMP Ctrl) complexed with lyovec, lyovec alone, 0.5 μM CpG2216 or 100 μg/mL pI:C for 18h. CD80 and CD86 expression on DC subsets was determined using flow cytometry. **(A)** Bar graphs represent the mean difference between geometric mean fluorescence intensities (gMFI) of stained samples and fluorescence minus one (FMO) controls ± SEM from 3 independent experiments. **(B)** Representative histograms showing CD80 and CD86 expression on all sorted DC subsets after stimulations. Grey unfilled lines represent FMO control and red filled lines represent Ab stain. Histograms represent 1 of 3 independent experiments. Statistical analyses were performed using two-tailed Paired Student’s t test where *P < 0.05 and ns, not significant.

Supplementary Figure 6 Type I and III IFNs are differentially produced by DC subsets after cGAMP stimulation. Sorted splenic mouse cDC1, cDC2 and pDCs from a pool of 15-17 mice were stimulated with 10 nmol 3’3’ cGAMP or its linearized control ligand (linGAMP Ctrl) complexed with lyovec, lyovec alone, 0.5 μM CpG2216 or 100 μg/mL pI:C for 18 hrs. IFN production in cell culture supernatants was analysed by ELISA (IFN-α and IFN-λ) or flow cytometric bead assay (IFN-β). Bar graphs represent mean ± SEM from 3 independent experiments. Statistical analyses were performed using two-tailed Paired Student’s t test where *P < 0.05 and ns, not significant.

Supplementary Figure 7 cDC subsets after cGAMP stimulation. FACS plots showing bulk mouse splenic cDC1 (CD11c^hi^CD317^lo^CD8^+^CD11b^lo^) and cDC2 (CD11c^hi^CD317^lo^CD8^-^CD11b^hi^) subsets stimulated for 18 h with 10 nmol 3’3’ cGAMP complexed with lyovec, or lyovec alone.

Supplementary Figure 8 cDC2 are partially killed after CDN stimulation. Bulk splenic DCs were stimulated with 1 or 10 nmol 2’3’ cGAMP, 3’3’ cGAMP, c-di-AMP or c-di-GMP complexed with lyovec, their respective linearized control ligands (Ctrl) complexed with lyovec, lyovec alone or 0.5 μM CpG2216 for 18h. Bar graphs represent the mean relative survival of cDC subsets ± SEM from 3 biological replicates (pool of 2 mice per replicate). Statistical analyses were performed using two-tailed Paired Student’s t test where *P < 0.05 and **P < 0.01 and ns, not significant.

Supplementary Figure 9 Caspase 3 is involved in cGAMP-mediated pDC death. **(A, B)** Bulk splenic DCs from BM chimeras generated using Ly5.2 WT or Vav-Cre Bax-/-/Bak-/- mice were stimulated with 10 nmol 3’3’ cGAMP or its linearized control ligand (linGAMP Ctrl) complexed with lyovec, lyovec alone or 0.5 μM CpG2216 for 18h. **(A)** Cytokine production in cell supernatants was analysed by ELISA (IFN-λ and IFN-α) or flow cytometric bead assay (TNF-α). Dotted lines represent upper limit of detection. Bar graphs show mean ± SEM from 2-3 individual mice per genotype. **(B)** Activation markers CD86 and CD69 were determined by flow cytometry. (i) Histograms show CD86 expression on pDCs. Dotted unfilled lines represent fluorescence minus one (FMO) control and solid filled lines represent Ab stain. Black and red lines represent WT and Vav-Cre Bax-/-/Bak-/- BM chimeric mice respectively. (ii) Bar graphs show the mean difference between geometric mean fluorescence intensities (gMFI) of stained samples and FMO controls ± SEM from 2-3 individual mice per genotype. **(C)** Bulk splenic DCs from a pool of 8-14 mice per replicate were treated with or without 5 μM pan-caspase inhibitor Q-VD-OPh for 1h before stimulations with 10 nmol 3’3’ cGAMP or its linearized control ligand (linGAMP Ctrl) complexed with lyovec or media alone for 4h. pDC numbers were enumerated using flow cytometry and bar graphs show mean relative survival (compared to media alone) ± SEM compiled from 3 independent experiments. **(D)** Sorted splenic mouse cDC1, cDC2 and pDCs (see **Supplementary Figure 4A** for sorting strategy) from a pool of 11-12 mice per replicate were stimulated with 10 nmol 3’3’ cGAMP or its linearized control ligand (linGAMP Ctrl) complexed with lyovec for 1.5 h. Cells were then lysed and blotted using antibodies specific for cleaved caspase 3 and actin. (i) Immunoblot shown represents 1 of 3 independent experiments. (ii) Densitometric analysis of cleaved caspase 3 relative to actin compiled from the immunoblots of 3 independent experiments. Bar graphs show mean ± SEM. **(E)** Sorted splenic mouse pDCs from a pool of 7-14 mice per replicate were stimulated with 10 nmol 3’3’ cGAMP or its linearized control ligand (linGAMP Ctrl) complexed with lyovec for 1.5 h. Caspase 3/7 activity was determined using Caspase-Glo 3/7^®^ Assay according to manufacturer’s instructions. Bar graphs show mean relative caspase 3/7 activity (compared to linGAMP Ctrl) ± SEM compiled from 2-3 independent experiments. Statistical analyses were performed using two-tailed Paired Student’s t test where *P < 0.05 and ns, not significant.

Supplementary Figure 10 Humanised mice DC are activated, but not killed, after cGAMP stimulation. Enriched, unsorted humanised mice DCs from BM were stimulated with 25 μg/mL pI:C, 10 μM CpG2216, 10 nmol 2’3’ cGAMP or its linearized control ligand complexed (linGAMP Ctrl) with lyovec for 18h. **(A)** Bar graphs represent the average change in CD80 gMFI values in stimulated samples compared to media only samples ± SEM from 3 independent experiments. **(B)** Bar graphs represent the mean relative survival (compared to media alone) ± SEM compiled from 3 independent experiments. **(C, D)** Human blood DC from 4 independent donors were stimulated with 5 nmol 2’3’ cGAMP or its linearized control ligand (linGAMP Ctrl) complexed with lyovec in the presence or absence of 5 μg/mL neutralising anti-IFN-λ1 antibody (anti-IL-29, MAB15981, R&D Systems) or antibody control for 18 h. **(C)** pDC numbers were enumerated using flow cytometry. Bar graphs show mean ± SEM and each dot represents individual human blood donors (n=4). **(D)** CD69 expression on pDC were determined using flow cytometry. Bar graphs shown mean difference between geometric mean fluorescence intensities (gMFI) of stained samples and FMO controls ± SEM and each dot represents individual human blood donors (n=4). Statistical analyses were performed using two-tailed Paired Student’s t test where *P < 0.05, **P < 0.01 and ns, not significant.
