# Supplementary figures and images for: Discordance in STING-Induced Activation and Cell Death Between Mouse and Human Dendritic Cell Populations
Source: Front Immunol. 2022 Feb 25;13:794776. doi: 10.3389/fimmu.2022.794776 (PMC8914948; doi:10.3389/fimmu.2022.794776)

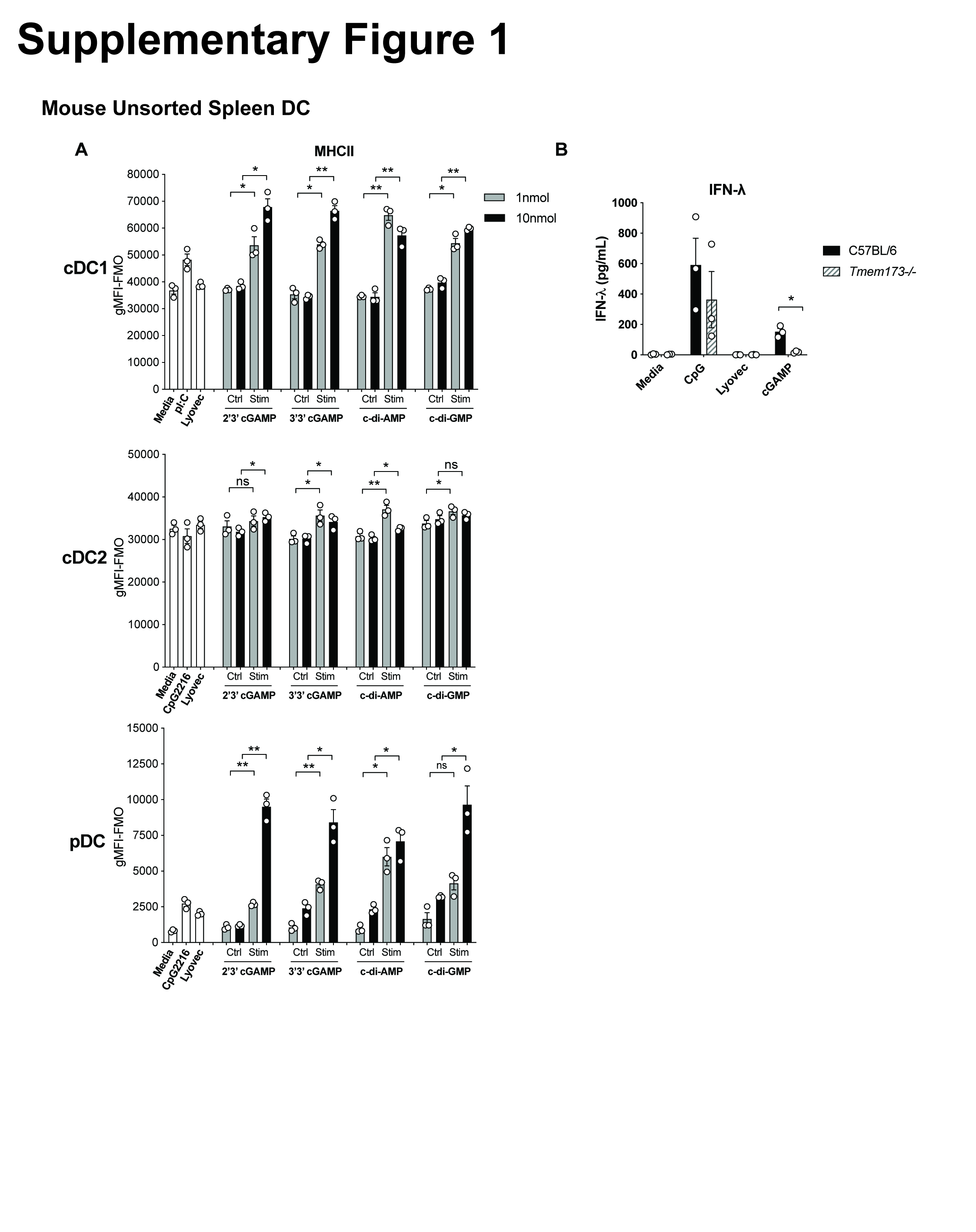

Supplement: Supplementary Figure 1 — Mouse DCs are potently activated by CDNs and produce IFN-λ that is STING-dependent. (A) Bulk splenic mouse DCs were stimulated with 1 or 10nmol 2’3’ cGAMP, 3’3’ cGAMP, c-di-AMP or c-di-GMP complexed with lyovec, their respective linearized control ligands (Ctrl) complexed with lyovec, lyovec alone, 0.5 µM CpG2216 or 100 µg/mL pI:C for 18h. MHCII expression on DC subsets was determined using flow cytometry. Bar graphs represent the mean difference between geometric mean fluorescence intensities (gMFI) of stained samples and fluorescence minus one (FMO) controls ± SEM from 3 biological replicates (pool of 2 mice per replicate). (B) Bulk splenic mouse DCs from C57BL/6 or Tmem173-/- were stimulated with 10nmol 3’3’ cGAMP complexed with lyovec, lyovec alone or 0.5 µM CpG2216 for 18h. IFN-λ production in cell culture supernatants were analysed by ELISA. Bar graphs represent mean ± SEM from 3 individual mice per genotype. Statistical analyses were performed using two-tailed Paired Student’s t test where *P < 0.05, ** P < 0.01and ns, not significant. [file Image_1.tif]

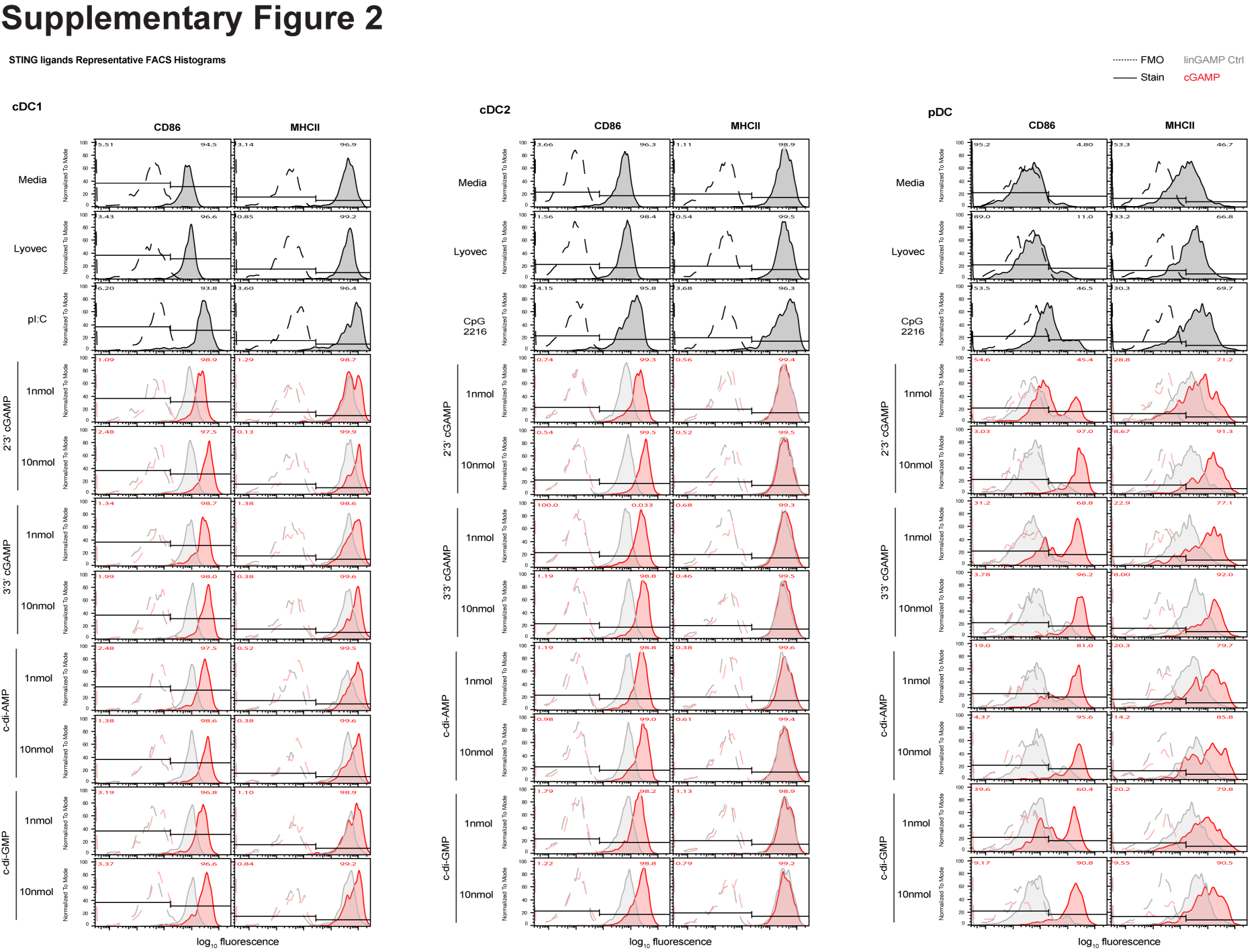

Supplement: Supplementary Figure 2 — Mouse DCs upregulate activation markers CD86 and MHCII after CDN stimulation. Bulk splenic mouse DCs were stimulated with 1 or 10nmol 2’3’ cGAMP, 3’3’ cGAMP, c-di-AMP or c-di-GMP complexed with lyovec, their respective linearized control ligands (linGAMP Ctrl) complexed with lyovec, lyovec alone, 0.5 µM CpG2216 or 100 µg/mL pI:C for 18h. Histograms show CD86 and MHCII expression on DC determined using flow cytometry. Dotted lines represent fluorescence minus one (FMO) control and filled in lines represent Ab stain. Grey lines represent linGAMP Ctrl and red lines represent cGAMP samples. Histograms represent 1 of 3 biological replicates (pool of 2 mice per replicate). [file Image_2.tif]

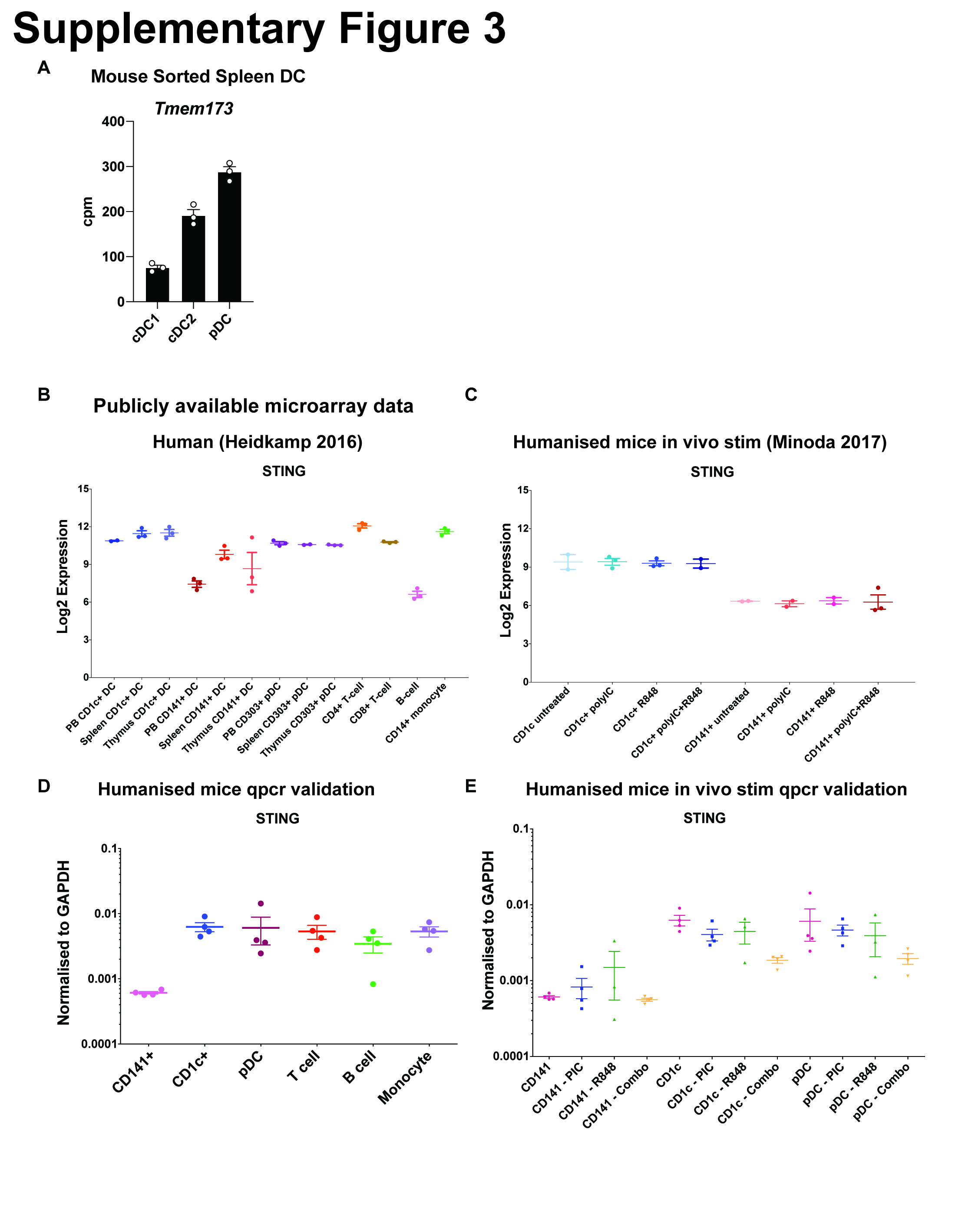

Supplement: Supplementary Figure 3 — STING transcripts in mouse and human DCs. (A) Tmem173 transcript expression (counts per million) from RNA-sequencing analysis of steady state sorted mouse splenic DCs. Bar graphs represent mean ± SEM from 3 independent samples of 10-14 mice pooled per sample. (B) Publicly available microarray data sets [www.stemformatics.org] represent expression levels of STING in human immune cells isolated from different organs (Heidkamp, 2016). (C) Microarray data demonstrating the changes in gene expression levels of STING in human cDCs when treated with polyI:C (TLR3 agonist), R848 (TLR7/8 agonist) and the combo (polyI:C+R848) (Minoda, 2017). (D, E) Quantitative PCR analysis was performed on immune cell subsets isolated from humanised mouse bone marrow to determine the pattern of STING expression in the steady state (D) as well as the expression levels after activation (E) in each DC subset. Gene expression levels were normalised to GAPDH. Each experiment was performed for 4 independent samples (n = 4) and the error bars represent mean ± SEM. [file Image_3.tif]

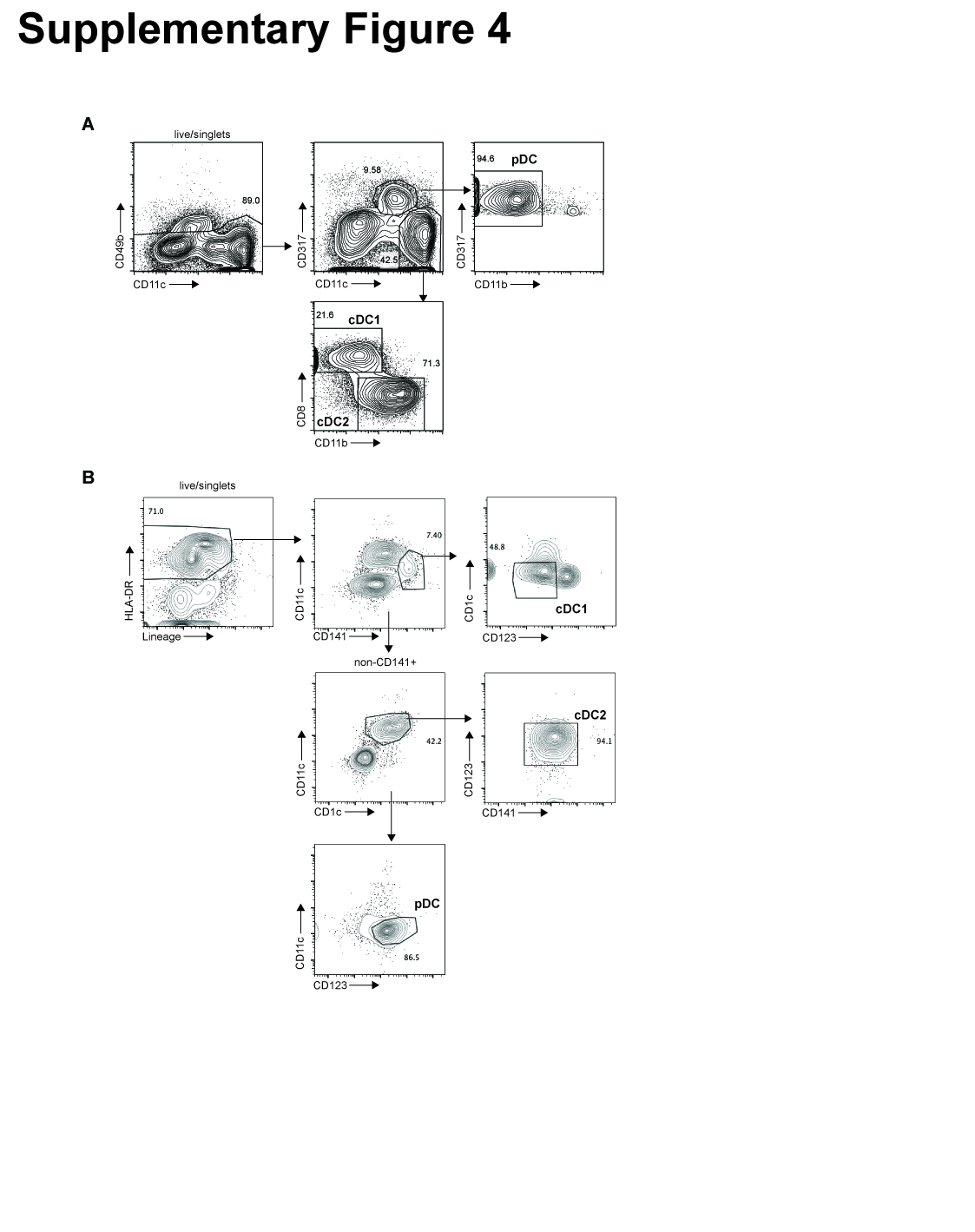

Supplement: Supplementary Figure 4 — Gating strategies for mouse and human DC subsets. (A) Bulk splenic DCs pooled from 15+17 C57BL/6 mice were labelled with the following fluorophore+conjugated mAbs: CD11c (N418), CD45RA (14.8), CD8 (53+6.7), CD11b (M1/70), CD49b (DX5). They were then sorted by first removing NK cells using junk gate and sorted into pDC (CD11cintCD317hiCD11b-), cDC1 (CD11chiCD317loCD8+CD11b-) and cDC2 (CD11chiCD317loCD8-CD11b-) subsets. (B) Purified human blood DCs were labelled with the following fluorophore+conjugated mAbs: CD1c (L161), CD3-ε (BC3), CD11c (B+ly6), CD14 (FMC17), CD16 (3G8), CD19 (FMC63), CD20 (B1), CD34 (AC133), CD57 (HNK1.1), CD69 (FN50), CD86 (IT2.2), CD123 (7G3), CD141 (AD5+14H12), Glycophorin A (10F7MN) and HLA+DR (REA332). DCs were first gated on HLA-DR+Lin- cells followed by separation into cDC1 (CD11c+CD141+CD1c-CD123-), cDC2 (CD11c+CD1c+) and pDC (CD11c-CD123+). [file Image_4.tif]

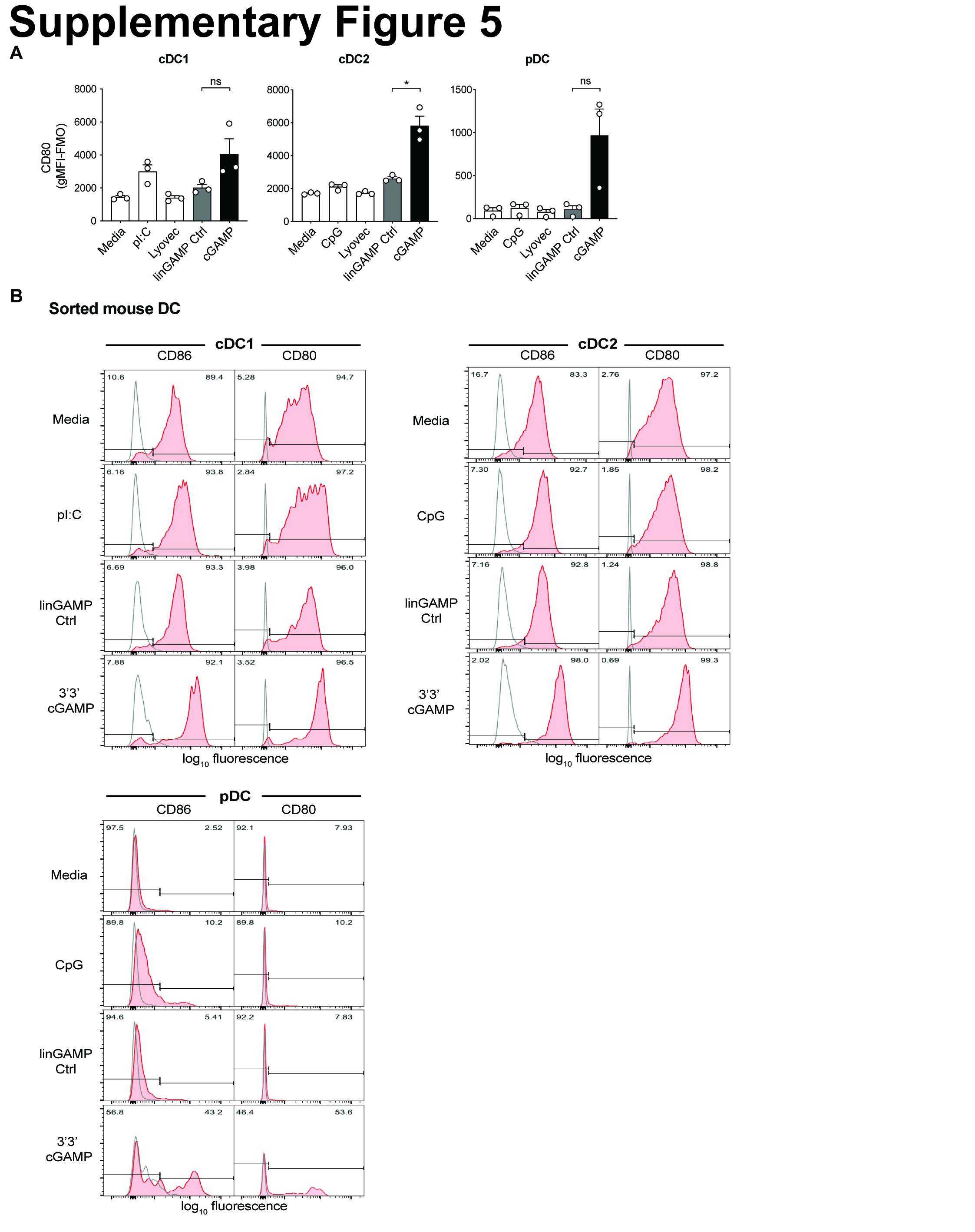

Supplement: Supplementary Figure 5 — Sorted mouse DCs upregulate CD80 and CD86 expression after cGAMP stimulation. Sorted splenic mouse cDC1, cDC2 and pDCs from a pool of 15-17 mice were stimulated with 10 nmol 3’3’ cGAMP or its linearized control ligand (linGAMP Ctrl) complexed with lyovec, lyovec alone, 0.5 μM CpG2216 or 100 μg/mL pI:C for 18h. CD80 and CD86 expression on DC subsets was determined using flow cytometry. (A) Bar graphs represent the mean difference between geometric mean fluorescence intensities (gMFI) of stained samples and fluorescence minus one (FMO) controls ± SEM from 3 independent experiments. (B) Representative histograms showing CD80 and CD86 expression on all sorted DC subsets after stimulations. Grey unfilled lines represent FMO control and red filled lines represent Ab stain. Histograms represent 1 of 3 independent experiments. Statistical analyses were performed using two-tailed Paired Student’s t test where *P < 0.05 and ns, not significant. [file Image_5.tif]

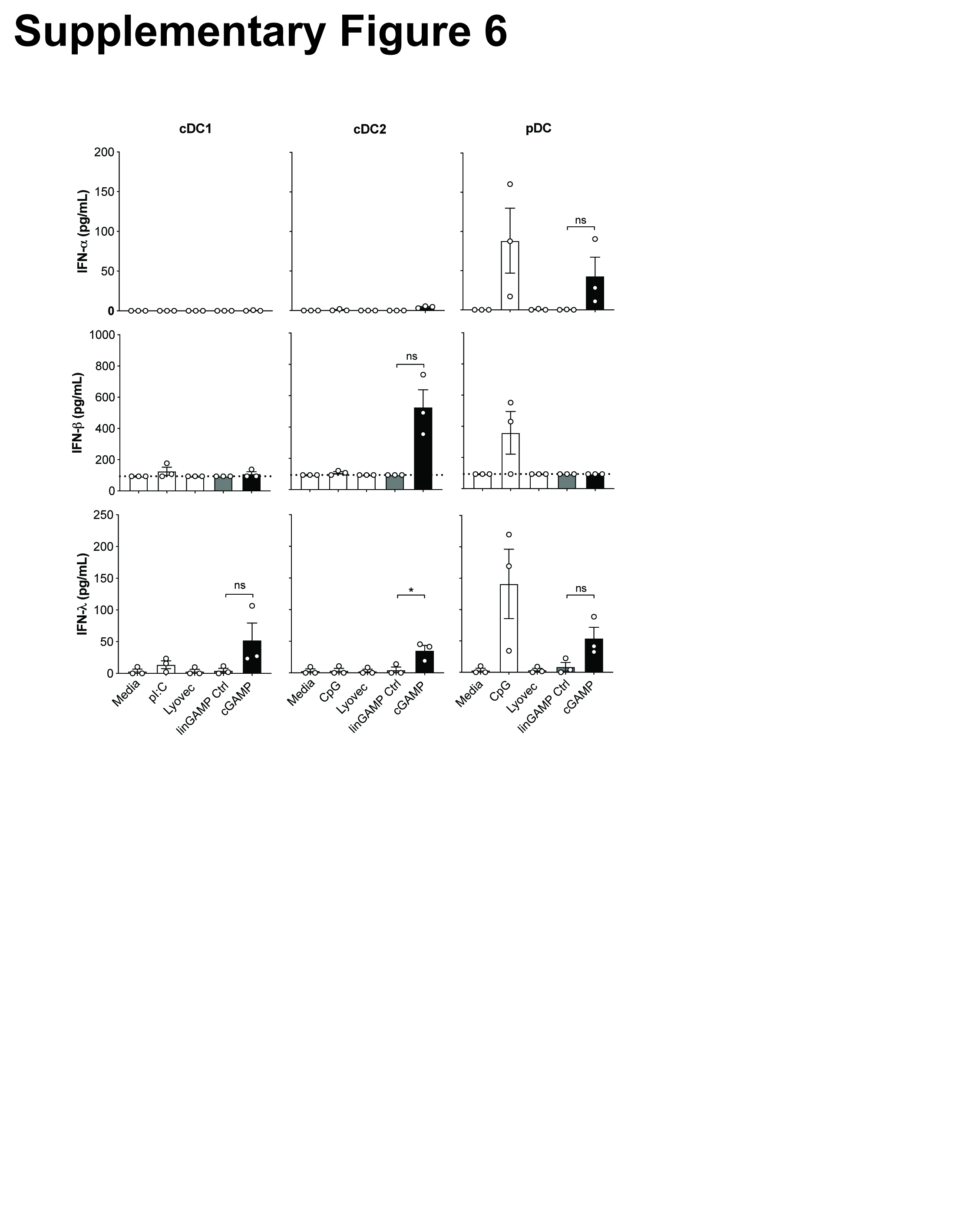

Supplement: Supplementary Figure 6 — Type I and III IFNs are differentially produced by DC subsets after cGAMP stimulation. Sorted splenic mouse cDC1, cDC2 and pDCs from a pool of 15-17 mice were stimulated with 10 nmol 3’3’ cGAMP or its linearized control ligand (linGAMP Ctrl) complexed with lyovec, lyovec alone, 0.5 μM CpG2216 or 100 μg/mL pI:C for 18 hrs. IFN production in cell culture supernatants was analysed by ELISA (IFN-α and IFN-λ) or flow cytometric bead assay (IFN-β). Bar graphs represent mean ± SEM from 3 independent experiments. Statistical analyses were performed using two-tailed Paired Student’s t test where *P < 0.05 and ns, not significant. [file Image_6.tif]

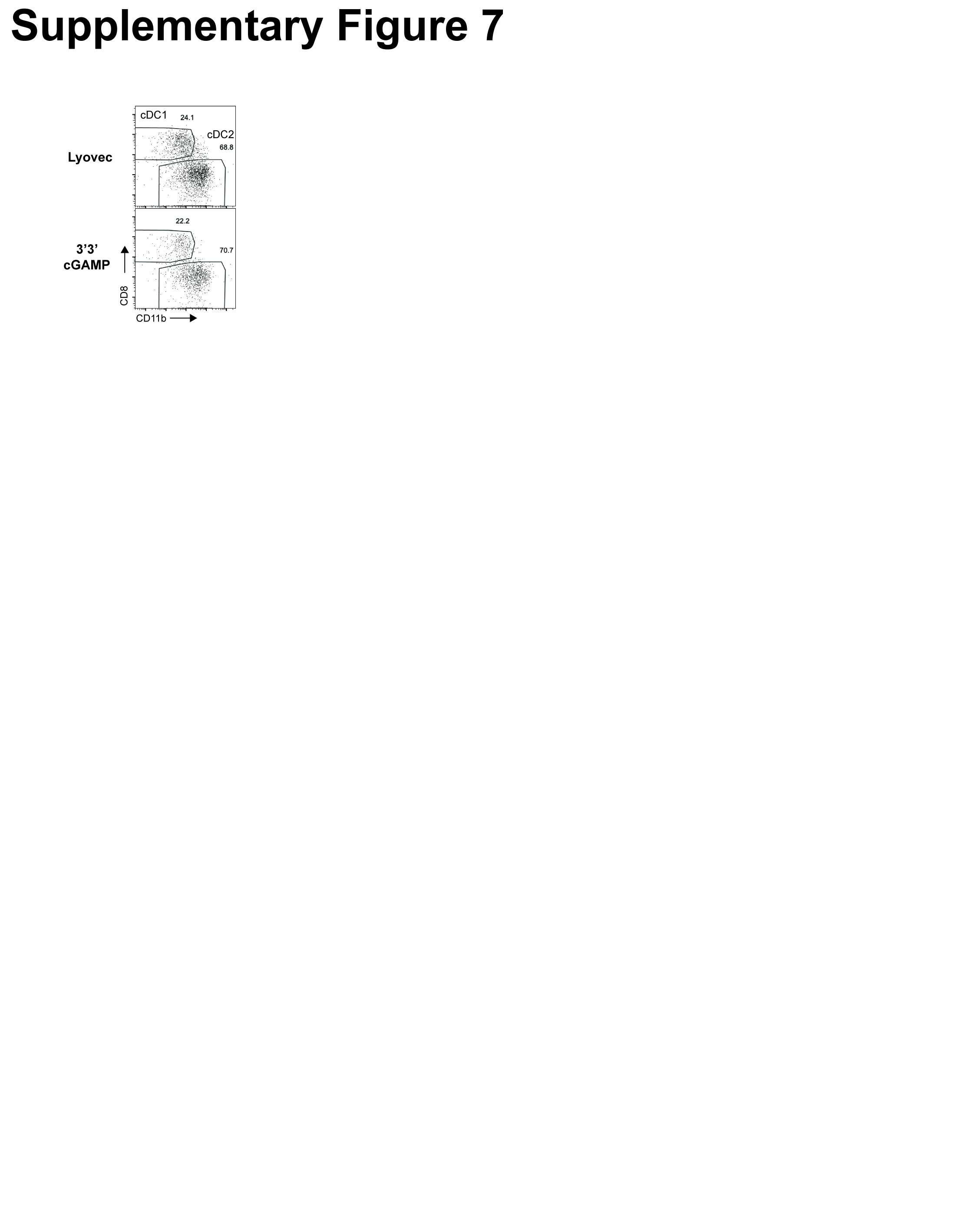

Supplement: Supplementary Figure 7 — cDC subsets after cGAMP stimulation. FACS plots showing bulk mouse splenic cDC1 (CD11chiCD317loCD8+CD11blo) and cDC2 (CD11chiCD317loCD8-CD11bhi) subsets stimulated for 18 h with 10 nmol 3’3’ cGAMP complexed with lyovec, or lyovec alone. [file Image_7.tif]

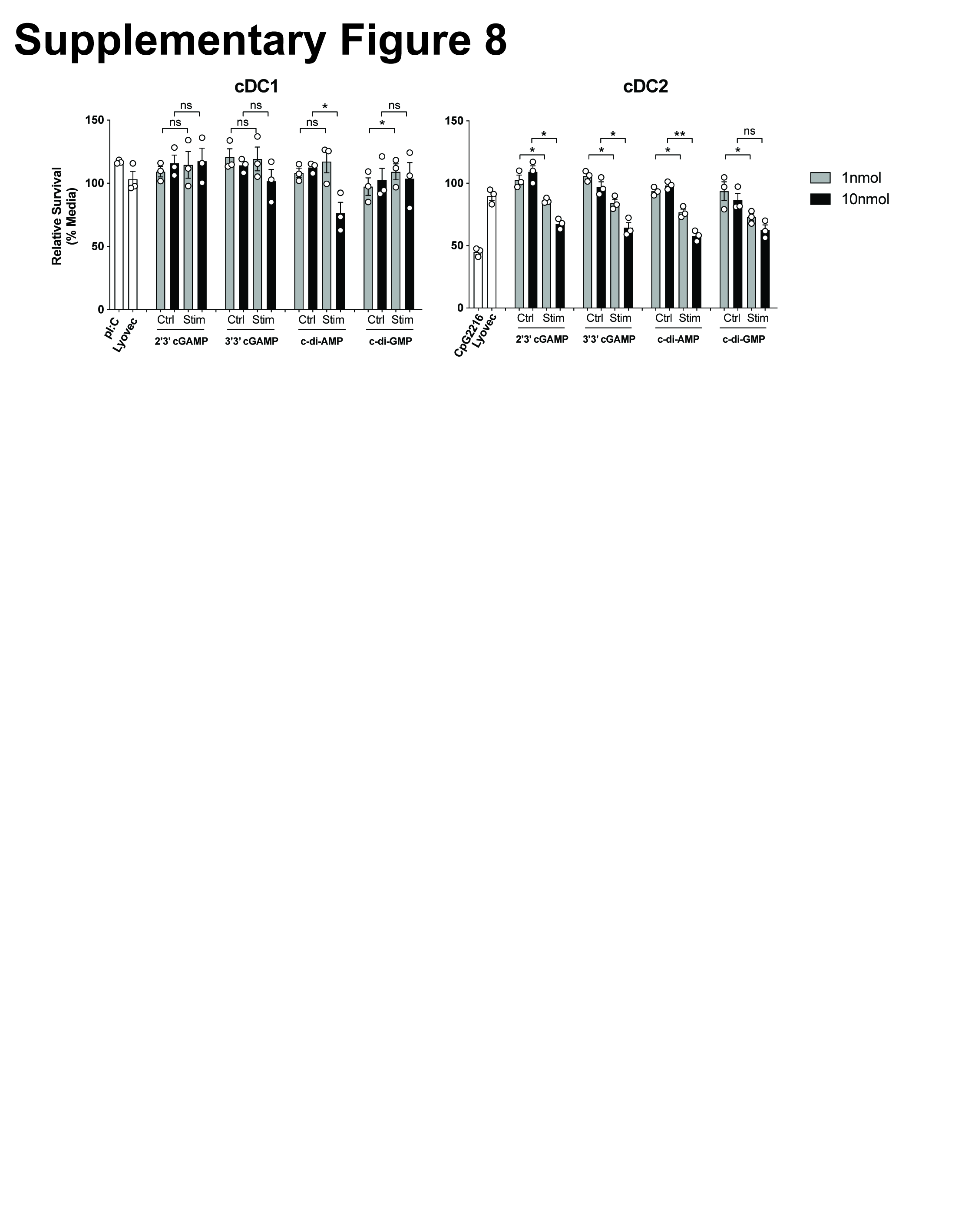

Supplement: Supplementary Figure 8 — cDC2 are partially killed after CDN stimulation. Bulk splenic DCs were stimulated with 1 or 10 nmol 2’3’ cGAMP, 3’3’ cGAMP, c-di-AMP or c-di-GMP complexed with lyovec, their respective linearized control ligands (Ctrl) complexed with lyovec, lyovec alone or 0.5 μM CpG2216 for 18h. Bar graphs represent the mean relative survival of cDC subsets ± SEM from 3 biological replicates (pool of 2 mice per replicate). Statistical analyses were performed using two-tailed Paired Student’s t test where *P < 0.05 and **P < 0.01 and ns, not significant. [file Image_8.tif]

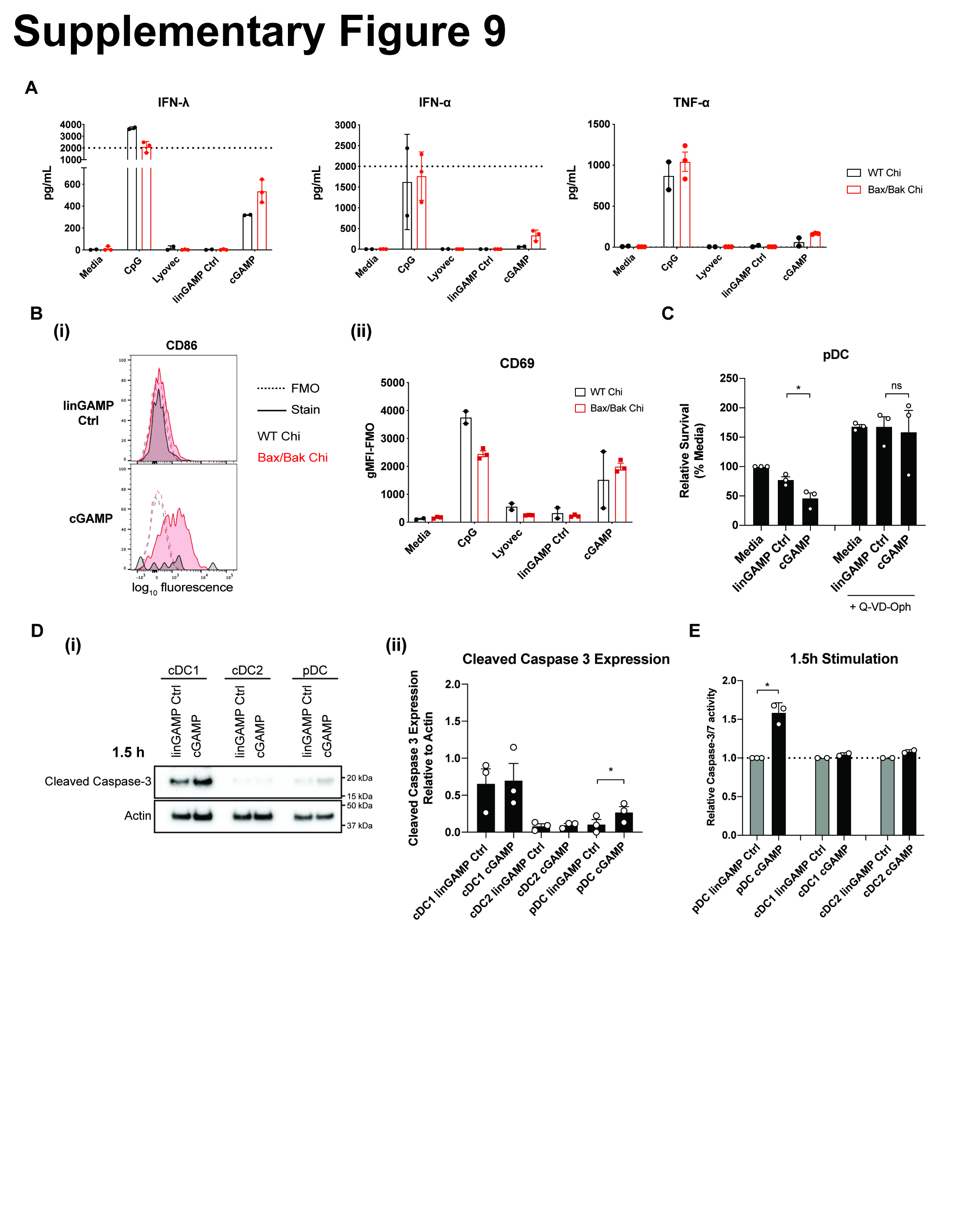

Supplement: Supplementary Figure 9 — Caspase 3 is involved in cGAMP-mediated pDC death. (A, B) Bulk splenic DCs from BM chimeras generated using Ly5.2 WT or Vav-Cre Bax-/-/Bak-/- mice were stimulated with 10 nmol 3’3’ cGAMP or its linearized control ligand (linGAMP Ctrl) complexed with lyovec, lyovec alone or 0.5 μM CpG2216 for 18h. (A) Cytokine production in cell supernatants was analysed by ELISA (IFN-λ and IFN-α) or flow cytometric bead assay (TNF-α). Dotted lines represent upper limit of detection. Bar graphs show mean ± SEM from 2-3 individual mice per genotype. (B) Activation markers CD86 and CD69 were determined by flow cytometry. (i) Histograms show CD86 expression on pDCs. Dotted unfilled lines represent fluorescence minus one (FMO) control and solid filled lines represent Ab stain. Black and red lines represent WT and Vav-Cre Bax-/-/Bak-/- BM chimeric mice respectively. (ii) Bar graphs show the mean difference between geometric mean fluorescence intensities (gMFI) of stained samples and FMO controls ± SEM from 2-3 individual mice per genotype. (C) Bulk splenic DCs from a pool of 8-14 mice per replicate were treated with or without 5 μM pan-caspase inhibitor Q-VD-OPh for 1h before stimulations with 10 nmol 3’3’ cGAMP or its linearized control ligand (linGAMP Ctrl) complexed with lyovec or media alone for 4h. pDC numbers were enumerated using flow cytometry and bar graphs show mean relative survival (compared to media alone) ± SEM compiled from 3 independent experiments. (D) Sorted splenic mouse cDC1, cDC2 and pDCs (see Supplementary Figure 4A for sorting strategy) from a pool of 11-12 mice per replicate were stimulated with 10 nmol 3’3’ cGAMP or its linearized control ligand (linGAMP Ctrl) complexed with lyovec for 1.5 h. Cells were then lysed and blotted using antibodies specific for cleaved caspase 3 and actin. (i) Immunoblot shown represents 1 of 3 independent experiments. (ii) Densitometric analysis of cleaved caspase 3 relative to actin compiled from the immunoblots of 3 independent exper [file Image_9.tif]

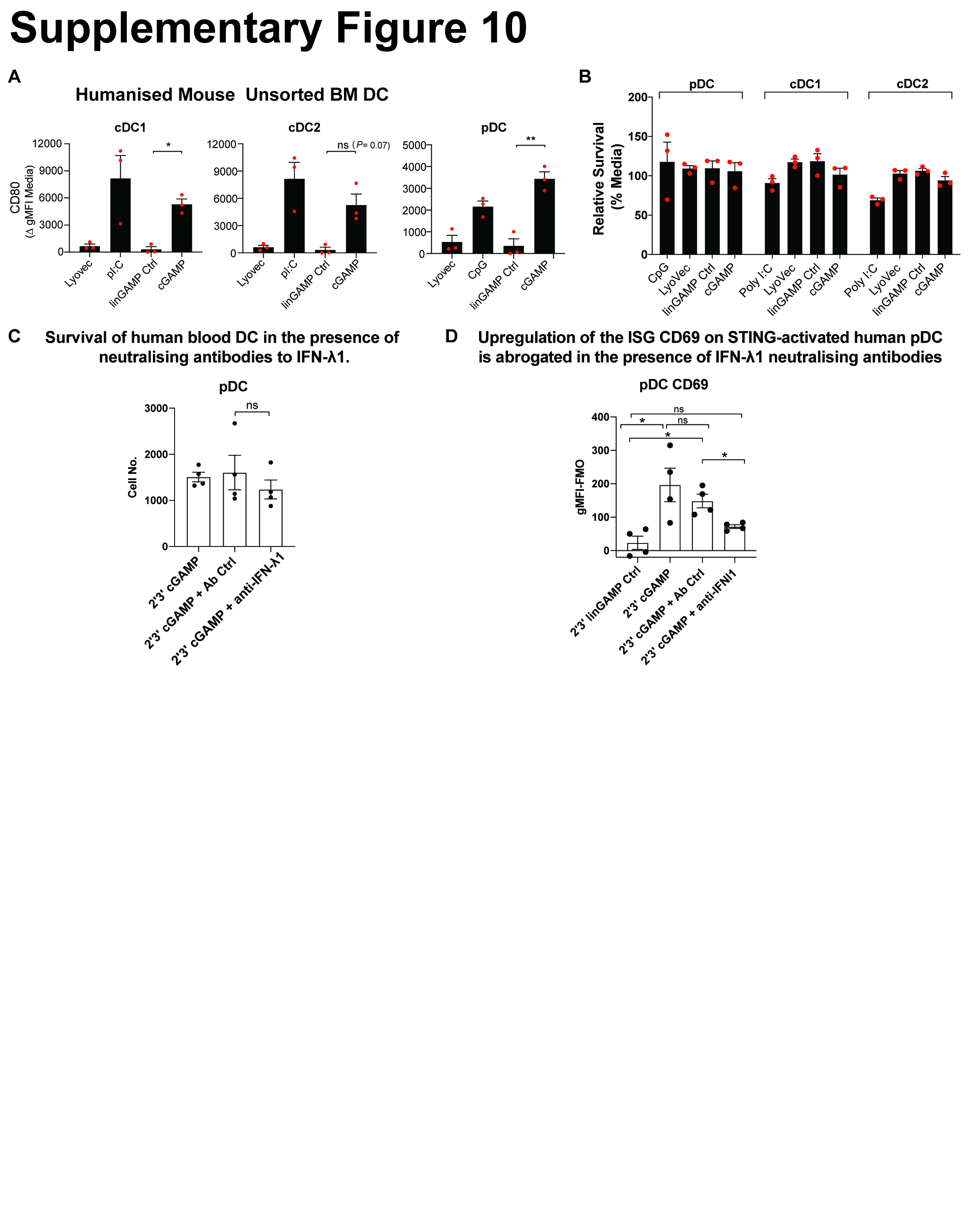

Supplement: Supplementary Figure 10 — Humanised mice DC are activated, but not killed, after cGAMP stimulation. Enriched, unsorted humanised mice DCs from BM were stimulated with 25 μg/mL pI:C, 10 μM CpG2216, 10 nmol 2’3’ cGAMP or its linearized control ligand complexed (linGAMP Ctrl) with lyovec for 18h. (A) Bar graphs represent the average change in CD80 gMFI values in stimulated samples compared to media only samples ± SEM from 3 independent experiments. (B) Bar graphs represent the mean relative survival (compared to media alone) ± SEM compiled from 3 independent experiments. (C, D) Human blood DC from 4 independent donors were stimulated with 5 nmol 2’3’ cGAMP or its linearized control ligand (linGAMP Ctrl) complexed with lyovec in the presence or absence of 5 μg/mL neutralising anti-IFN-λ1 antibody (anti-IL-29, MAB15981, R&D Systems) or antibody control for 18 h. (C) pDC numbers were enumerated using flow cytometry. Bar graphs show mean ± SEM and each dot represents individual human blood donors (n=4). (D) CD69 expression on pDC were determined using flow cytometry. Bar graphs shown mean difference between geometric mean fluorescence intensities (gMFI) of stained samples and FMO controls ± SEM and each dot represents individual human blood donors (n=4). Statistical analyses were performed using two-tailed Paired Student’s t test where *P < 0.05, **P < 0.01 and ns, not significant. [file Image_10.tif]
